# Aerobic exercise improves astrocyte mitochondrial quality and transfer to neurons in a mouse model of Alzheimer's disease

**DOI:** 10.1111/bpa.13316

**Published:** 2024-10-26

**Authors:** Jiachen Cai, Yan Chen, Yuzhu She, Xiaoxin He, Hu Feng, Huaiqing Sun, Mengmei Yin, Junying Gao, Chengyu Sheng, Qian Li, Ming Xiao

**Affiliations:** ^1^ Jiangsu Key Laboratory of Neurodegeneration Nanjing Medical University Nanjing China; ^2^ Nanjing Brain Hospital Affiliated to Nanjing Medical University Nanjing China; ^3^ Department of Neurology The First Affiliated Hospital of Nanjing Medical University Nanjing China; ^4^ Department of Anatomy Nanjing Medical University Nanjing China

**Keywords:** Alzheimer's disease, astrocytes, CD38, exercise, mitochondrial transfer

## Abstract

Mitochondrial dysfunction is a well‐established hallmark of Alzheimer's disease (AD). Despite recent documentation of transcellular mitochondrial transfer, its role in the pathogenesis of AD remains unclear. In this study, we report an impairment of mitochondrial quality within the astrocytes and neurons of adult 5 × FAD mice. Following treatment with mitochondria isolated from aged astrocytes induced by exposure to amyloid protein or extended cultivation, cultured neurons exhibited an excessive generation of reactive oxygen species and underwent neurite atrophy. Notably, aerobic exercise enhanced mitochondrial quality by upregulating CD38 within hippocampal astrocytes of 5 × FAD mice. Conversely, the knockdown of CD38 diminished astrocytic–neuronal mitochondrial transfer, thereby abolishing the ameliorative effects of aerobic exercise on neuronal oxidative stress, β‐amyloid plaque deposition, and cognitive dysfunction in 5 × FAD mice. These findings unveil an unexpected mechanism through which aerobic exercise facilitates the transference of healthy mitochondria from astrocytes to neurons, thus countering the AD‐like progression.

## Graphical abstract

Aerobic exercise improves transfer of healthy mitochondria from astrocytes to neurons in the 5 × FAD mice.

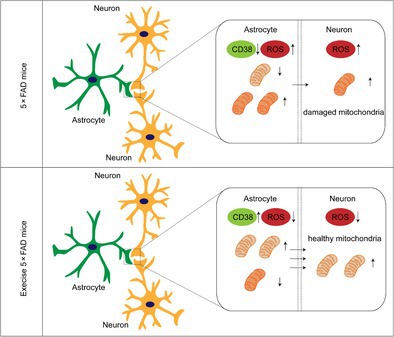

## 1 INTRODUCTION

The brain is a unique organ with the highest energy metabolism in the human body. Impaired brain energy metabolism serves as an early pathological manifestation of neurodegenerative diseases, including Alzheimer's disease (AD) [1, 2]. Mitochondria not only generate ATP but also act as the primary producers of intracellular reactive oxygen species (ROS) and are susceptible to oxidative stress caused by various toxic metabolites, such as β‐amyloid (Aβ) [3, 4]. Only a minute fraction of mitochondria undergoes senescence under physiological conditions [5]. The aged mitochondria are sequestered within autophagosomes and subsequently transferred to lysosomes for degradation, while newly synthesized mitochondria are promptly regenerated [6]. Recent evidence suggests that mitochondrial intercellular transfer also plays a crucial role in the maintenance of mitochondrial homeostasis [7, 8]. The aberrant mitochondrial exchange between cells has been implicated in the pathogenesis of several neurological diseases including stroke and acute respiratory distress syndrome [9, 10, 11].

Astrocytes, as the most abundant cells in the brain, are tasked with regulating cerebral blood flow, material metabolism, and energy supply [12]. A mounting body of evidence supports the notion that mitochondrial transfer between astrocytes and neurons represents a novel target for neuroprotection. For instance, in a mouse model of stroke, functional mitochondria can be transferred from astrocytes to adjacent neurons, mitigating neuronal damage [13, 14]. Notably, 5 × FAD mice exhibit abnormal neuron–astrocyte transmitophagy, characterized by an increased degradation of neuronal mitochondria by astrocytes [15]. Mitochondrial damage in astrocytes can lead to the accumulation of lipid droplets, thereby further exacerbating the pathological progression of AD [16, 17, 18]. Consequently, enhancing the quality of astrocyte mitochondria and bolstering neighboring neurons may constitute a novel strategy to delay or even prevent the onset of AD. Nevertheless, the means to achieve this goal and the underlying mechanisms remain to be determined.

Exercise emerges as one of the most pragmatic and secure strategies to mitigate the aging process and reduce the risk of AD [19]. Aerobic exercise has been demonstrated to be an effective measure for combating muscle aging by enhancing mitochondrial quality [20]. Exercise promotes the upregulation of mitochondrial peroxisome proliferator‐activated receptor gamma coactivator 1‐alpha, a principal regulator of mitochondrial biogenesis, within muscle cells [21]. Nevertheless, it currently remains uncertain whether aerobic exercise can improve the quality of astrocyte mitochondria and facilitate mitochondrial transfer during the neurodegenerative process of AD.

Recent reports have unveiled that CD38 signaling is responsible for the extracellular release of mitochondria by astrocytes in a mouse model of stroke [13]. During exercise, CD38 in skeletal muscle is upregulated through the cAMP‐responsive element‐binding protein (CREB) pathway [22]. CD38 is highly enriched in astrocytes, which regulates their development and activation [13, 23]. Nevertheless, the variations in CD38 expression in astrocytes during AD and the role of aerobic exercise in modulating CD38 expression in astrocytes remain enigmatic.

In this study, we sought to ascertain whether mitochondrial quality in hippocampal astrocytes and neurons, as well as astrocyte–neuronal transfer, is perturbed in 5 × FAD transgenic mice. We endeavored to determine whether these alterations could be rectified through sustained aerobic exercise. Additionally, we explored whether aerobic exercise mitigates AD‐like pathology through the CD38‐mediated transfer of healthy mitochondria from astrocytes to neurons. In vitro experiments were also conducted to appraise the impact of mitochondria secreted by aged astrocytes on the maintenance of neurites in cultured neurons. These findings unveil a novel mechanism through which aerobic exercise combats AD.

## 2 RESULTS

### 2.1 Poor quality of astrocytic mitochondria that enter neurons in 5 × FAD mice

**FIGURE 1:**
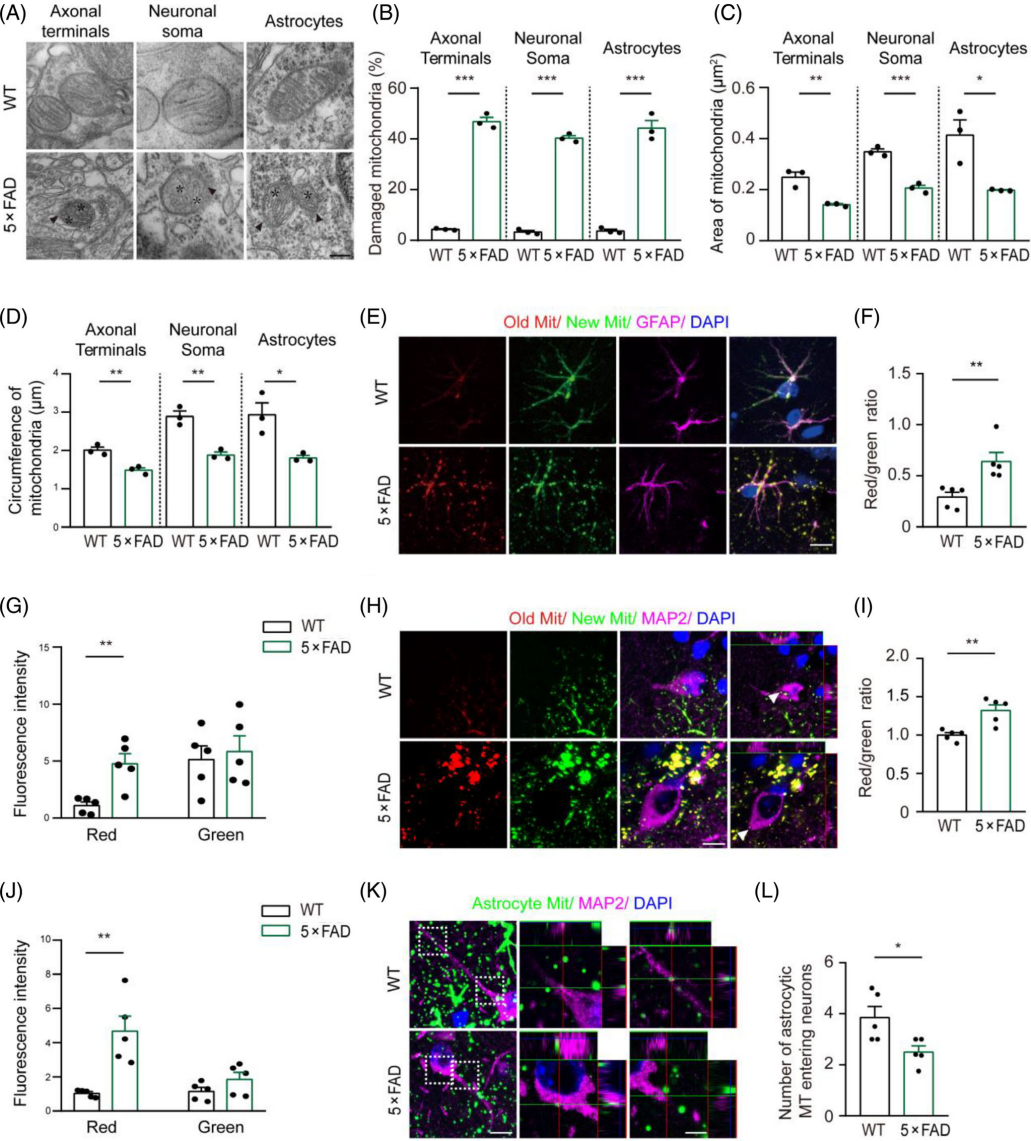
Decreased mitochondrial quality in the hippocampus of 6‐month‐old 5 × FAD mice. (A) Representative images of mitochondria in the hippocampal axon terminals, neuronal soma, and astrocytes of wild‐type (WT) and 5 × FAD mice. Swollen or disrupted mitochondrial cristae are indicated by asterisks, while broken mitochondrial membranes are indicated by arrows. Scale bar: 200 nm. (B) Quantification of the percentage of abnormal mitochondria in (A). *n* = 3 mice, 30 mitochondria per mouse for each index. (C, D) Quantification of the area and circumference of the mitochondria in (A). *n* = 3 mice, 30 mitochondria per mouse for each index. (E) Representative images of astrocytic mitochondria labeled by AAV‐glial fibrillary acidic protein (GFAP)‐mito‐timer virus and GFAP antibody in the hippocampal astrocytes of WT and 5 × FAD mice. Scale bar: 10 μm. (F) Quantification of the ratio of red (old mitochondria)/green (new mitochondria) fluorescence intensities in (E). *n* = 5 mice, 30 GFAP‐positive cells per mouse. (G) Quantification of red (old mitochondria) and green (new mitochondria) fluorescence intensity in (E). *n* = 5 mice, 30 GFAP‐positive cells per mouse. (H) Representative images of microtubule‐associated protein 2 (MAP2) and astrocytic mitochondria labeled by AAV‐GFAP‐mito‐timer entering hippocampal neurons of WT and 5 × FAD mice. Scale bar: 10 μm. (I) Quantification of the ratio of the red/green fluorescence intensity in (H). *n* = 5 mice, 10 MAP2‐positive neurons per mouse. (J) Quantification of red (old mitochondria) and green (new mitochondria) fluorescence intensity in (H). (K) Representative images of MAP2 and astrocytic mitochondria labeled by AAV‐GFAP‐mito‐timer entering hippocampal neurons of WT and 5 × FAD mice. Scale bar: 10 μm and 2.5 μm (high magnification images). (L) Quantification of the number of hippocampal astrocytic mitochondria of WT and 5 × FAD mice entering neurons in (K). *n* = 5 mice, 10 MAP2‐positive neurons per mouse. Paired *t*‐test. Data are presented as mean ± SEM. *p < 0.05, **p < 0.01, ***p < 0.001.

It is well known that mitochondrial structural damage and secondary oxidative damage have been implicated in the pathological process of AD [24, 25, 26]. Transmission electron microscopy confirmed this view and revealed that 6‐month‐old 5 × FAD mice had varying degrees of abnormalities in the mitochondrial ultrastructure in hippocampal neuronal soma, axonal terminals, and astrocytes, manifested as condensed matrix, swollen cristae, and/or broken mitochondrial membrane (Figure 1A). In contrast, in hippocampal neurons and astrocytes of age‐matched wild‐type (WT) mice, the vast majority of mitochondria exhibited an intact ultrastructure (Figure 1A). Statistical results showed that in neuronal bodies, axon terminals, and astrocytes of AD mice, more than 40% of mitochondrial ultrastructure was impaired, and mitochondrial area and circumference were decreased (Figure 1B–D). Consistently, ROS levels were significantly elevated in the hippocampal neuron‐enriched CA region in 5 × FAD mice compared with the WT littermates (Figure S1A,B). Further analysis demonstrated that the expression of superoxide dismutase 2 (SOD2) decreased in hippocampal neurons and astrocytes of 5 × FAD mice (Figure S1C–F). Together, these data confirm that mitochondrial oxidative damage occurs in hippocampal neurons and astrocytes under AD‐like pathological conditions.

Recent findings suggest that mitochondria in astrocytes can be transported to adjacent neuronal soma or synaptic structures [13]. We evaluated the quality of astrocyte mitochondria in 5 × FAD mice by injecting the AAV‐glial fibrillary acidic protein (GFAP)‐mito‐timer virus. The mitotic timer protein used in this study is a mutant of the red fluorescent protein dsRed, whose fluorescence irreversibly changes from green to red because of the oxidation of Tyr67 residues [27]. The increase in the ratio of red/green fluorescence emission intensity indicates a decrease in mitochondrial protein turnover ability and can reflect the deepening of mitochondrial aging, thus enabling the detection of mitochondrial quality [28]. The results showed that in 5 × FAD mice, the mitochondrial quality markedly decreased in hippocampal astrocytes (Figure 1E–G). Furthermore, when compared with WT mice, the quality and number of astrocytic mitochondria that entered adjacent neurons were also reduced in 5 × FAD mice (Figure 1H–L). These data together reveal impaired structure and function of astrocytic mitochondria in the AD mice. Aging astrocyte mitochondria that enter neurons might exacerbate oxidative damage to neurons.

### 2.2 Mitochondria secreted from Aβ‐treated astrocytes intensified atrophy and oxidative stress of cultured neurons

**FIGURE 2:**
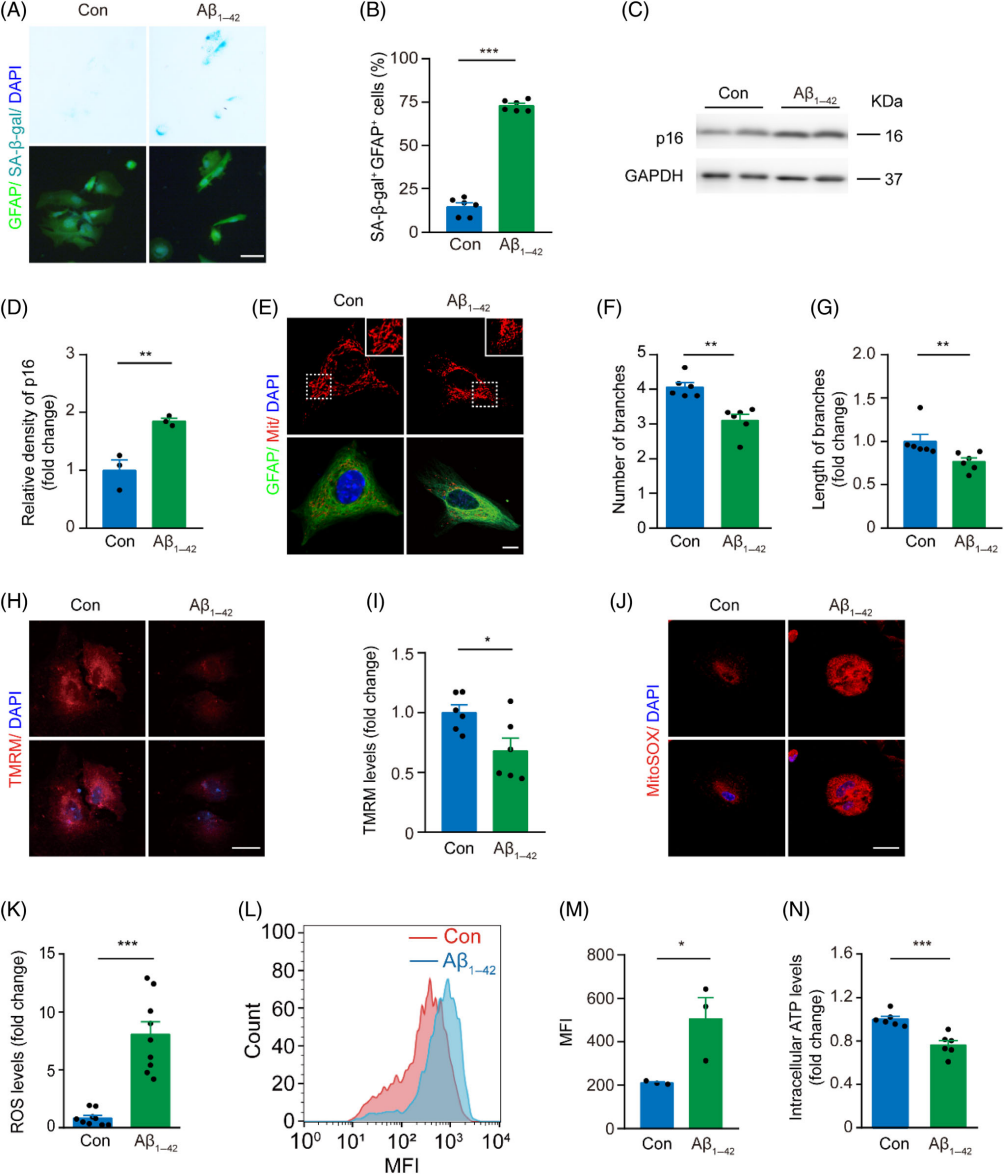
Impaired mitochondria in Aβ_1–42_‐treated primary astrocytes. (A) Representative images of control (Con) and Aβ_1–42_‐treated primary astrocytes labeled with SA‐β‐gal and glial fibrillary acidic protein (GFAP). Scale bar: 50 μm. (B) Quantification of the percentage of SA‐β‐gal^+^GFAP^+^ cells. *n* = 6 samples, 50 GFAP‐positive cells per sample. (C, D) Western blot and densitometry analysis of protein levels of p16 from control (Con) and Aβ_1–42_‐treated astrocytes. *n* = 3 samples, triple repeats. (E) Representative images of GFAP and mitochondria labeled by MitoTracker Red CMXRos in control (Con) and Aβ_1–42_‐treated astrocytes. Scale bar: 50 μm. (F, G) Quantification of mitochondrial branch number and branch length. *n* = 6 samples, 20 GFAP‐positive cells per sample. (H, I) Representative images and quantification of TMRM in control (Con) and Aβ_1–42_‐treated astrocytes. *n* = 6 samples, 20 TMRM‐positive cells per sample. Scale bar: 10 μm. (J, K) Representative images and quantification of intracellular reactive oxygen species (ROS) production in control (Con) and Aβ_1–42_‐treated astrocytes. *n* = 9 samples, five fields per sample. Scale bar: 20 μm. (L, M) Flow cytometry and quantification analysis of Mito‐SOX fluorescence intensity (MFI), a highly selective indicator of ROS in live cell mitochondria, in control (Con) and Aβ_1–42_‐treated astrocyte. *n* = 3 samples. (N) Intracellular ATP levels in control (Con) and Aβ_1–42_‐treated astrocytes. *n* = 6 samples. Paired *t*‐test. Data are presented as mean ± SEM. *p < 0.05, **p < 0.01, ***p < 0.001. Aβ, β‐amyloid.

To validate our hypothesis, we conducted a series of in vitro experiments. Primary astrocytes were isolated from the hippocampus of newborn mice and cultured for 2 weeks before treatment with 5 μM Aβ_1–42_ oligomers for 24 h. The Aβ‐treated primary astrocytes exhibited a significantly higher percentage of positive SA‐β‐gal staining, compared with untreated astrocytes (Figure 2A,B). In line with this, Western blot analysis revealed elevated expression levels of p16, a marker of cellular senescence [29], in astrocytes treated with Aβ_1–42_ (Figure 2C,D). Mitochondrial Mitotracker staining, combined with GFAP immunofluorescence, revealed reductions in both the number and length of mitochondrial branches in astrocytes exposed to Aβ_1–42_ (Figure 2E–G). The toxic impact of Aβ_1–42_ on cultured astrocytes was further confirmed by a reduction in mitochondrial membrane potential (Figure 2H,I) and an increase in mitochondrial ROS levels (Figure 2J–M). Astrocytes exposed to Aβ_1–42_ resulted in decreased ATP levels (Figure 2N), further indicating their mitochondrial dysfunction.

**FIGURE 3:**
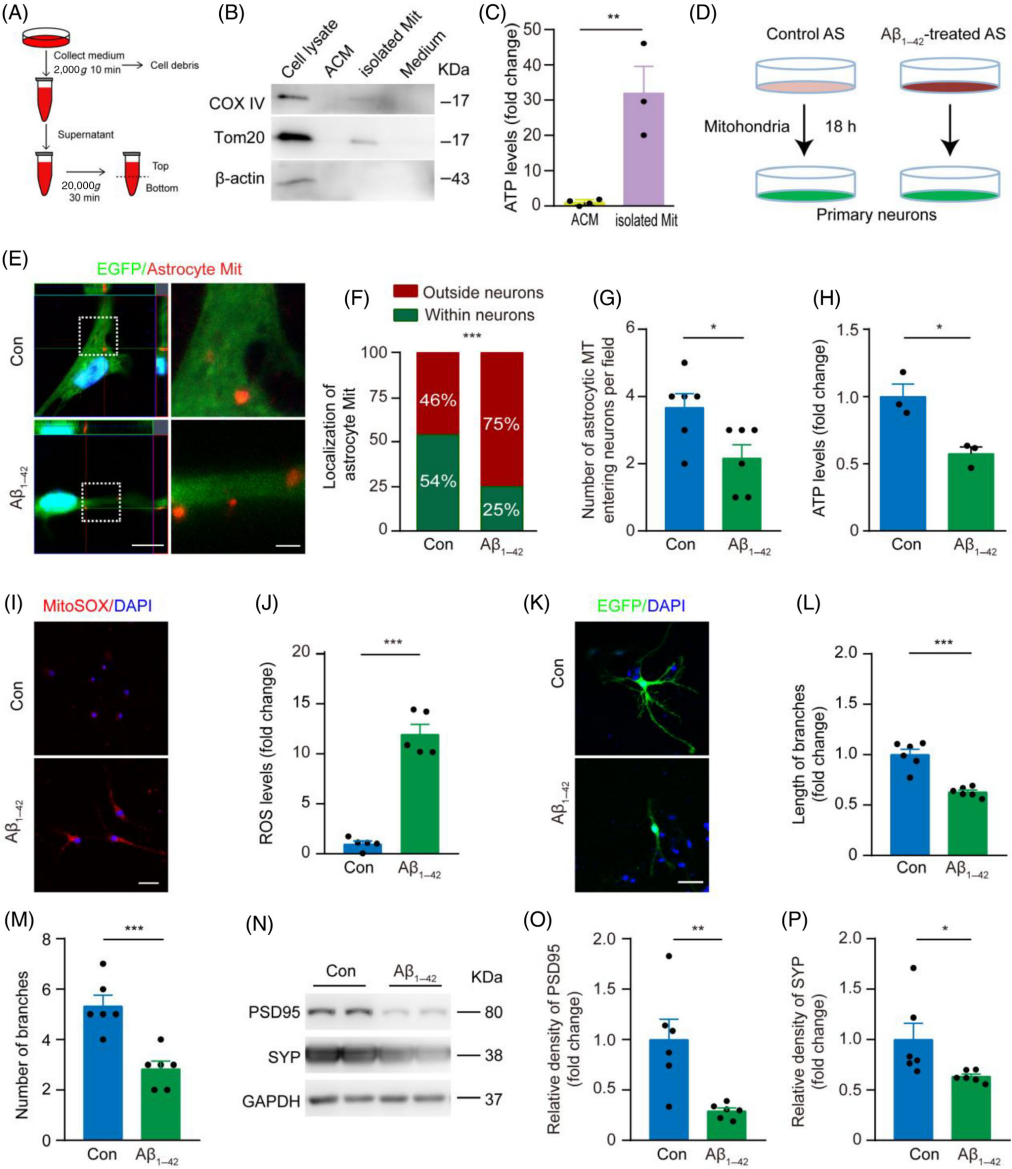
Aβ_1–42_‐treated astrocytic mitochondria entered neurons, increased reactive oxygen species (ROS) levels, and inhibited neuronal growth. (A) Schematic of mitochondrial extraction from astrocyte‐conditioned medium (ACM). (B) Western blotting for two mitochondrial markers COX IV and TOM20 in the mitochondria (Mit) isolated from ACM (80 mL per dish). ACM is the conditioned culture supernatant of astrocytes. Medium represents the fresh culture medium that has not yet been added to the culture dish. (C) Extracellular ATP in ACM and isolated mitochondria. *n* = 4 samples, triple repeats. (D) Schematic of cultured primary neurons treated with astrocytic mitochondria. (E, F) Representative images and percentage of control and Aβ_1–42_‐treated astrocytic mitochondria (red) entering cultured primary neurons. Scale bar: 10 μm and 5 μm (high magnification images). *n* = 3 samples, 20 EGFP positive cells per sample. (G) Quantification of the number of mitochondria from control and Aβ‐treated astrocyte entering neurons per field (44.94 μm^2^) of view. *n* = 6 samples. (H) Quantification of ATP levels in neurons treated with mitochondria isolated from Aβ‐treated astrocytes. *n* = 3 samples, triple repeats. (I, J) Representative images and quantification of intracellular ROS production of cultured primary neurons treated with mitochondria released by control and Aβ_1–42_‐treated astrocytes. Scale bar: 50 μm. *n* = 5 samples, 10 images per sample. (L, M) Representative images and quantification of neuronal branch length and branch number. Scale bar: 50 μm. *n* = 5 samples, 10 neurons per sample. (N–P) Western blot and densitometry analysis of protein levels of PSD95 and SYP of cultured primary neurons treated with mitochondria isolated from control and Aβ_1–42_‐treated astrocytes. *n* = 6 samples. Paired *t*‐test (C, G, H, J, L, M, O, P) or Chi‐square test (F). Data are presented as mean ± SEM. *p < 0.05, **p < 0.01, ***p < 0.001. Aβ, β‐amyloid.

Furthermore, we demonstrated the extracellular release of mitochondria by astrocytes by successfully detecting the mitochondrial markers cytochrome *c* oxidase IV (COX IV) and translocase of outer mitochondrial membrane 20 (TOM20) and ATP levels in the culture medium (Figure 3A–C). To explore whether mitochondria released from astrocytes could be transferred to neurons, we collected mitochondria labeled with Mitotracker from the supernatant of astrocytes with or without exposure to Aβ_1–42_. We found that Aβ_1–42_‐treated astrocytes released fewer mitochondria and had lower mitochondrial SOD activity (Figure S2A–D). Then, we co‐cultured astrocyte‐derived mitochondria with primary neurons that had received a prior transfection of HBLV‐ZsGreen for 18 h (Figure 3D). The mitochondria labeled with Mitotracker from the supernatant of primary astrocytes were detected in the neurons. The proportion and number of astrocyte‐derived mitochondria entering cultured neurons in the Aβ_1–42_‐treated group were less than those in the control group (Figure 3E–G). However, cultured neurons incubated with mitochondria from Aβ_1–42_ exposed astrocytes exhibited low ATP levels (Figure 3H), high ROS levels (Figure 3I,J), decreases in the number and length of neurite branches (Figure 3K–M), and low levels of synaptophysin (SYP) and post‐synaptic density protein 95 (PSD‐95) (Figure 3N–P), indicating the occurrence of atrophy and/or degeneration.

In order to exclude the toxic effects of small amounts of Aβ_1–42_ residues in astrocyte mitochondrial isolates on neurons and to further validate the negative impact of aging astrocyte mitochondria entering neurons, we utilized a long‐term culture‐induced astrocyte aging model. Most astrocytes underwent senescence after 8 weeks of culture with high expression levels of SA‐β‐gal and p16, compared with those cultured for 2 weeks (Figure S3A–D). Mitochondria derived from aged astrocytes displayed abnormal morphology (Figure S3E–G), decreased membrane potential (Figure S3H,I), and increased ROS levels (Figure S3J,K). The transfer of aged astrocyte‐derived mitochondria to primary cultured neurons elevated neuronal ROS levels (Figure S4A,B), diminished ATP content (Figure S4C), and reduced neurite length and branching (Figure S4D–F), although transfer efficiency was reduced when compared with mitochondria secreted by astrocytes cultured for 2 weeks (Figure S4G,H). These in vitro data further underscored the transfer of mitochondria released by aged astrocytes to neurons, subsequently resulting in oxidative damage.

### 2.3 Aerobic exercise improved the quality of astrocytic mitochondria and their transfer to neurons in 5 × FAD mice

**FIGURE 4:**
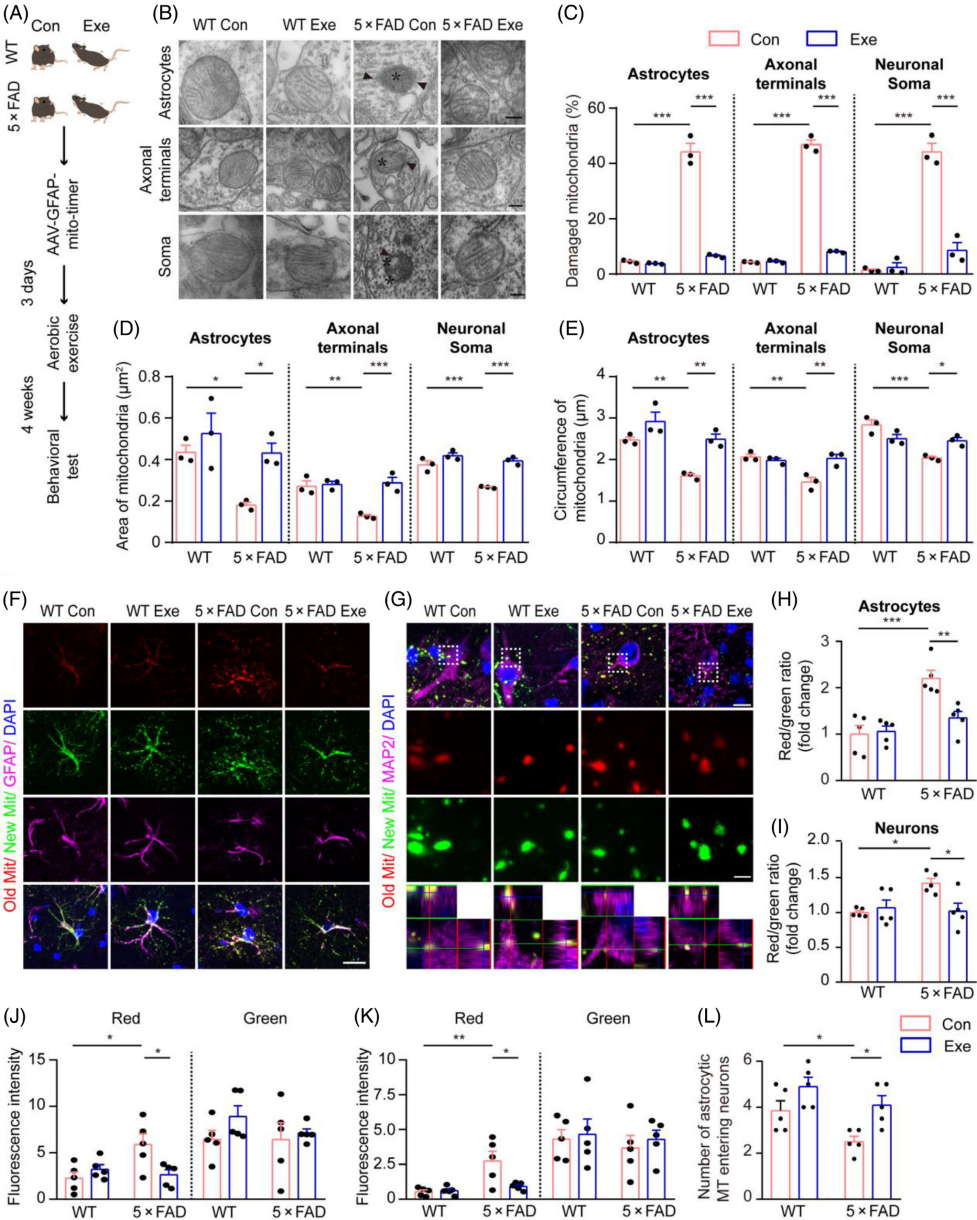
Enhancement of mitochondrial quality in hippocampal astrocytes of 6‐month‐old 5 × FAD mice by aerobic exercise. (A) Experimental schematic. (B) Representative images of mitochondria in the hippocampi of wild‐type (WT) and 5 × FAD mice. Swollen or disrupted mitochondrial cristae are indicated by asterisks, and broken mitochondrial membranes by arrows. Scale bar: 200 nm. (C) Quantification of the percentage of abnormal mitochondria in (B). *n* = 3 mice, 30 mitochondria per mouse for each index. (D, E) Quantification of the area and circumference of the mitochondria in (B). *n* = 3 mice, 30 mitochondria per mouse for each index. (F) Representative images of glial fibrillary acidic protein (GFAP) and mitochondria labeled by AAV‐GFAP‐mito‐timer in hippocampal astrocytes of WT and 5 × FAD mice. Scale bar: 20 μm. (G) Representative images of microtubule‐associated protein 2 (MAP2) and astrocytic mitochondria labeled by AAV‐GFAP‐mito‐timer entering hippocampal neurons of WT and 5 × FAD mice. Scale bar: 10 and 2.5 μm (high magnification images). (H) Quantification of the red/green fluorescence intensities ratio in (F). *n* = 5 samples, 30 GFAP‐positive astrocytes per sample. (I) Quantification of the red/green fluorescence intensities ratio in (G). *n* = 5 samples, 10 MAP2‐positive neurons per sample. (J) Quantification of red (old mitochondria) and green (new mitochondria) fluorescence intensities in (F). *n* = 5 samples, 30 GFAP‐positive astrocytes per sample. (K) Quantification of red (old mitochondria) and green (new mitochondria) fluorescence intensities in (G). *n* = 5 samples, 10 MAP2‐positive neurons per sample. (L) Quantification of the number of hippocampal astrocytic mitochondria of WT mice and 5 × FAD mice entering neurons in (E). *n* = 5 samples, 10 MAP2‐positive neurons per sample. Two‐way ANOVA, Tukey's multiple comparison test. Data are presented as the mean ± SEM. *p < 0.05, **p < 0.01, ***p < 0.001.

It has been recently reported that aerobic exercise enhances mitochondrial quality [30]. We evaluated the mitochondrial quality of astrocytes and their translocation to neurons by bilaterally injecting the AAV‐GFAP‐mito‐timer virus into the hippocampus of 5‐month‐old 5 × FAD mice following aerobic exercise for 4 weeks (Figure 4A). Transmission electron microscopy showed that exercised 5 × FAD mice had a reduced proportion of damaged mitochondria in neurons and astrocytes, accompanied with increased mitochondrial area and circumference (Figure 4B–E). The mitochondrial quality was notably enhanced in hippocampal astrocytes, including those entering adjacent neurons in 5 × FAD mice with exercise training, compared with their sedentary counterparts (Figure 4F–L). Western blot analysis further revealed decreases in the expression levels of p62, PINK1, and LC3, the markers of autophagic vacuoles [31], in the hippocampus of 5 × FAD mice that received exercise training (Figure S5A–D), further suggesting an improvement in mitochondrial quality. Additionally, aerobic exercise reduced ROS levels, increased SOD2 levels in neurons and astrocytes, and decreased Aβ accumulation of the hippocampus of 5 × FAD mice (Figure S6A–J). Results from behavioral tests demonstrated that aerobic exercise enhanced the cognitive performance of 5 × FAD mice in both the novel object recognition test and the Y‐maze test (Figure S7A–C). Collectively, these results suggest that aerobic exercise promotes the entry of healthy mitochondria from astrocytes into neurons, potentially providing benefits in alleviating AD‐like pathology in 5 × FAD mice.

### 2.4 Aerobic exercise promoted the entry of astrocytic mitochondria into neurons through the CD38 signaling pathway

**FIGURE 5:**
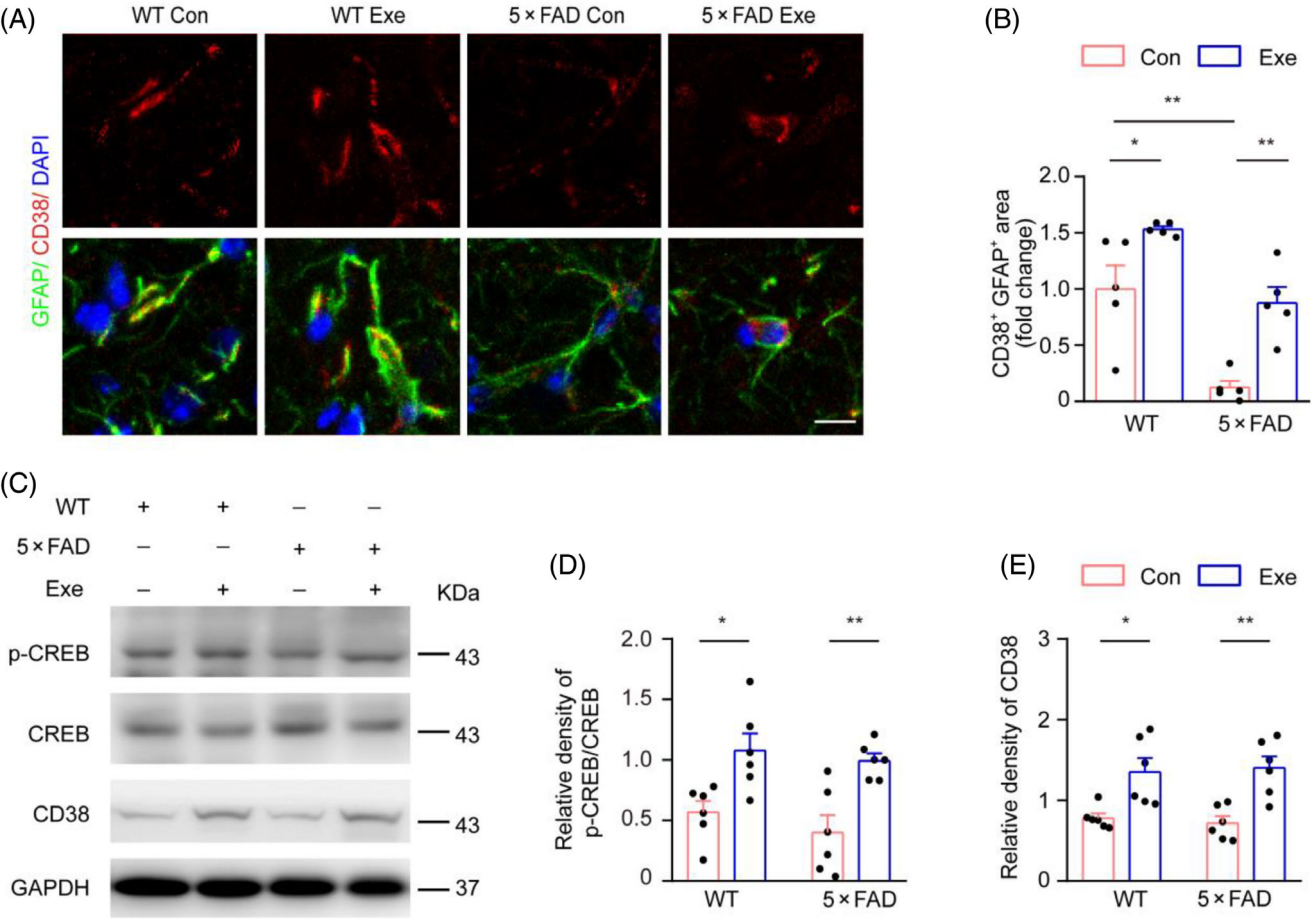
Upregulation of CD38 expression in hippocampal astrocytes of 6‐month‐old 5 × FAD mice by aerobic exercise. (A, B) Representative images of glial fibrillary acidic protein (GFAP) and CD38 staining and the area of CD38^+^GFAP^+^ signals in hippocampal astrocytes of wild‐type (WT) and 5 × FAD mice. Scale bar: 20 μm. *n* = 5 samples, 10 images per sample. (C–E) Western blot and densitometry analysis of protein levels of CD38 in the hippocampus of WT mice and 5 × FAD mice. *n* = 6 samples, three repeats. Two‐way ANOVA, Tukey's multiple comparison test. Data are presented as the mean ± SEM. *p < 0.05, **p < 0.01.

Recent studies have reported that the CD38 signaling pathway mediates the transfer of mitochondria from astrocytes to neurons [13]. CD38 is highly enriched in astrocytes, which regulates their development and activation [13, 23]. In order to validate this notion, we downregulated CD38 gene expression in primary astrocytes using CD38 short interfering RNA (siRNA). We found that the release of mitochondria was reduced in cultured astrocytes after knockdown of CD38 expression (Figure S8A–C). Then, we treated primary neurons with astrocyte‐derived mitochondria for 18 h. The results indicated a significant reduction in the number of astrocytic mitochondria entering neurons after CD38 knockdown (Figure S8D,E). We also observed decreases in CD38 expression in cultured astrocytes after exposure to Aβ_1–42_ oligomers (Figure S8F–I). Immunofluorescence results also demonstrated that the expression level of CD38 in hippocampal astrocytes of 5 × FAD mice was lower than in WT mice, and aerobic exercise upregulated CD38 expression in hippocampal astrocytes in both WT and AD mice (Figure 5A,B). Western blotting further confirmed the upregulation of CD38 expression and its transcriptional regulator CREB in the hippocampus of the both mouse genotypes after aerobic exercise (Figure 5C–E).

**FIGURE 6:**
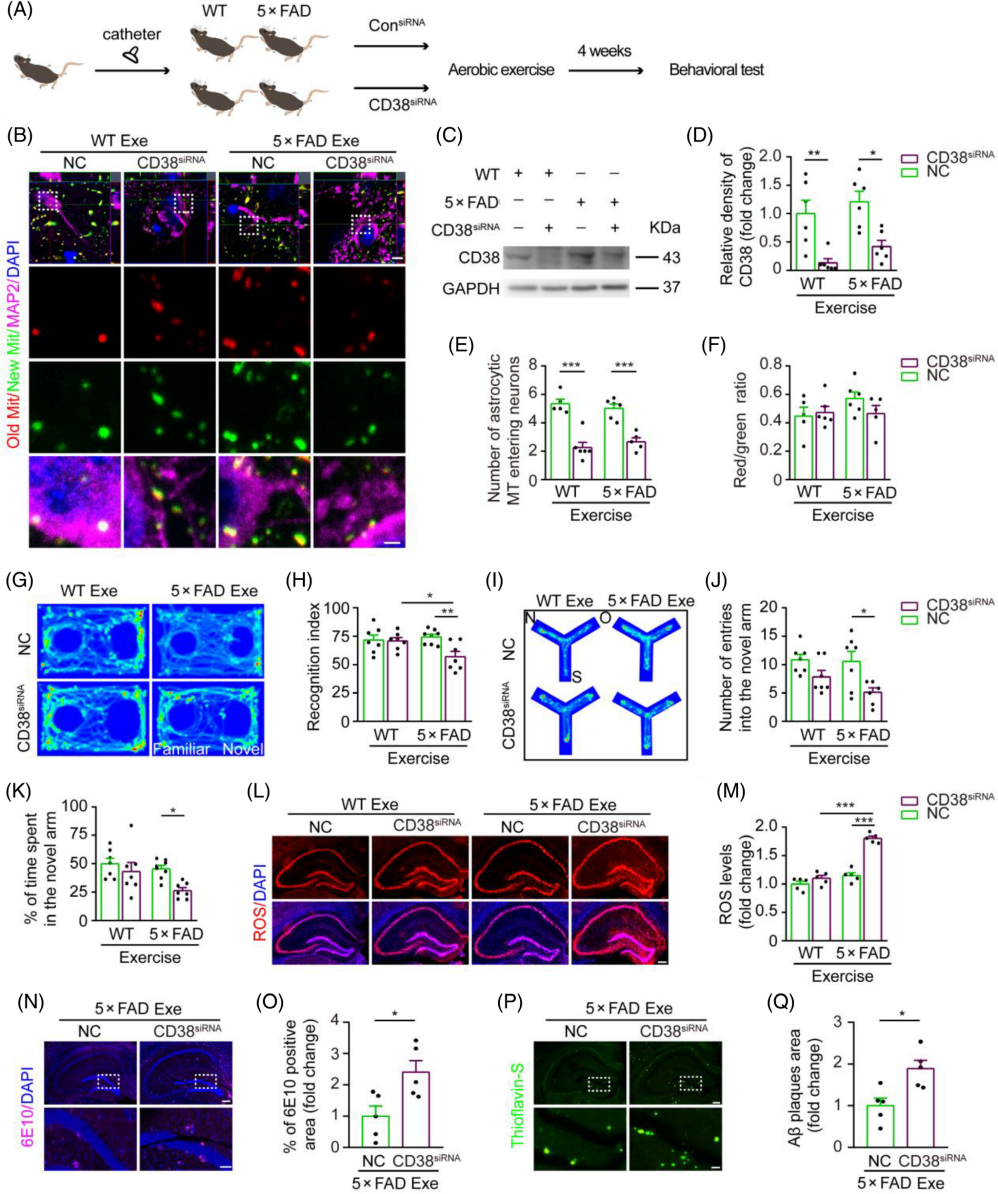
Inhibition of astrocytic mitochondrial entry into neurons and aggravation of Alzheimer's disease (AD)‐like phenotype in 6.5‐month‐old 5 × FAD mice receiving aerobic exercise after CD38 knockdown. (A) Experimental diagram. (B) Representative images of astrocytic mitochondria labeled by AAV‐glial fibrillary acidic protein‐mito‐GFP virus entering hippocampal neurons stained by microtubule‐associated protein 2 (MAP2) of wild‐type (WT) and 5 × FAD mice treated with negative control (NC) siRNA and CD38 siRNA. Scale bar: 5 μm and 1 μm (high magnification images). (C, D) Western blot and densitometry analysis of CD38 expression (*n* = 6 samples, three repeats). (E) The number of hippocampal astrocytic mitochondria entering neurons of WT and 5 × FAD mice treated with NC‐siRNA and CD38 siRNA. *n* = 5 mice, 15 MAP2 neurons per mouse. (F) Quantification of the red/green fluorescence intensities ratio in (B). *n* = 6 samples, 10 MAP2‐positive neurons per sample. (G) Movement tracing during the novel object recognition test (NORT). (H) Recognition index of NORT (*n* = 7–8 mice). (I) Movement trace in the Y‐maze (N: Novel arm; O: Old arm; S: Start arm). (J, K) Number of entries in the novel arm and percentage of time spent in the novel arm (*n* = 7–8 mice). (L, M) Representative images and quantification of reactive oxygen species (ROS) production in the hippocampus. Scale bar: 200 μm. *n* = 5 mice, 5 sections per mouse. (N, O) Representative images and quantification of 6E10^+^ plaques in the hippocampus. Scale bar: 200 and 50 μm (high magnification images). *n* = 5 mice, 5 sections per mouse. (P, Q) Representative images and quantification of thioflavin‐S^+^ plaques in the hippocampus. Scale bar: 200 and 50 μm (high magnification images). *n* = 5 mice, 5 sections per mouse. Two‐way ANOVA with Tukey's multiple comparison test (D–F, H, J, K, M) or paired *t*‐test (O, Q). Data are presented as the mean ± SEM. *p < 0.05, **p < 0.01, ***p < 0.001.

To determine whether the facilitation of astrocyte‐derived mitochondrial transfer to adjacent neurons by aerobic exercise depends on the CD38 signaling pathway, we injected CD38 siRNA into the bilateral hippocampus of 6‐month‐old WT and 5 × FAD mice that had previously received an injection of AAV‐GFAP‐mito‐timer virus. These animals were then subjected to 4 weeks of aerobic exercise training, followed by behavioral and pathological analyses (Figure 6A). The results revealed a decrease in the number of astrocytic mitochondria entering hippocampal neurons after CD38 knockdown in both WT mice and 5 × FAD mice that had undergone aerobic exercise (Figure 6B–E), while the quality of astrocytic mitochondria entering neurons was unchanged (Figure 6F). The novel object recognition test and the Y‐maze test indicated a decrease in cognitive function in CD38‐siRNA 5 × FAD mice, compared with NC‐siRNA controls (Figure 6G–K). We also found that hippocampal mitochondrial ROS levels and Aβ load increased in exercise‐5 × FAD mice after CD38 knockdown (Figure 6L–Q). Additionally, we injected con siRNA and CD38 siRNA into the hippocampus of WT mice without aerobic exercise, respectively, and found that knockdown of CD38 expression also inhibited astrocyte mitochondrial transport to neurons under physiological conditions (Figure S9A,B). Together, these results suggest that blocking the CD38 signaling pathway impedes the transfer of astrocytic mitochondria into neurons, thereby damaging the ameliorating effect of aerobic exercise on AD‐like pathology in 5 × FAD mice.

## 3 DISCUSSION

**FIGURE 7:**
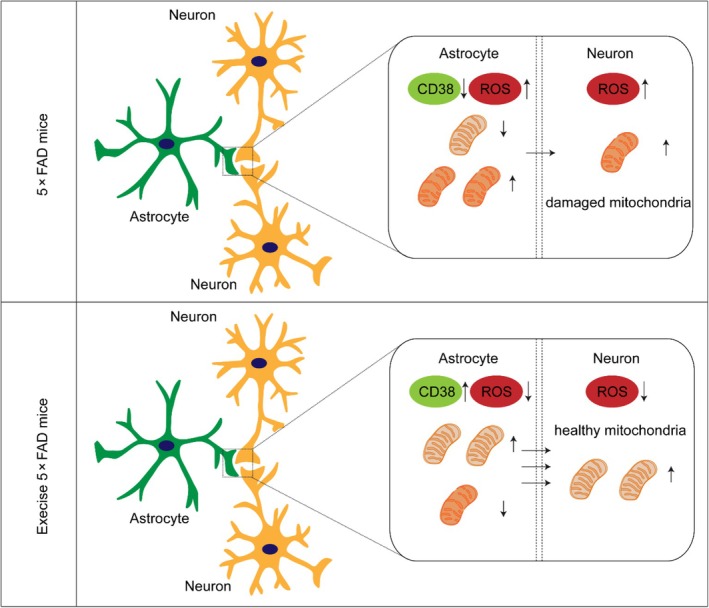
Illustration of the neuroprotective impact of aerobic exercise. The impaired mitochondrial transfer between astrocytes and neurons in 5 × FAD mice, characterized by diminished astrocytic mitochondrial quality and reduced CD38 expression, is improved by aerobic exercise. This enhancement increases the transfer of healthy astrocytic mitochondria to adjacent neurons, consequently lowering hippocampal oxidative stress levels and enhancing cognitive function in Alzheimer's disease (AD) mice. ROS, reactive oxygen species.

Mitochondrial dysfunction represents a hallmark of aging and age‐related neurodegeneration, including AD [32]. Protecting mitochondrial function is considered one of the most promising prevention and treatment strategies for AD. However, the effective methods to achieve this goal warrant further exploration. In this study, we observed a decrease in mitochondrial quality in hippocampal astrocytes and neurons of 6‐month‐old 5 × FAD mice. Aerobic exercise not only enhances mitochondrial quality, but also increases astrocytic–neuronal mitochondrial transfer by upregulating the CD38 signaling pathway. Inhibiting CD38 in the hippocampus hampers astrocytic mitochondrial transfer to neurons, thereby negating the ameliorating effect of aerobic exercise on oxidative stress and Aβ deposition in AD mice (Figure 7).

Neurons are highly reliant on mitochondrial ATP production, necessitating a constant renewal of mitochondria to maintain their survival and functionality [33, 34]. Neuronal mitochondrial dysfunction accelerates Aβ and Tau‐related pathological cascades in AD [35]. Unlike the dominant neurotransmission role of neurons, astrocytes primarily function to maintain brain homeostasis including energy supply and resistance to oxidative damage [36]. In response to various pathogenic stimuli, astrocytes undergo activation and serve neuroprotective roles [37]. Notably, reactive astrogliosis precedes neuronal degeneration in the AD progression [38]. However, persistently activated astrocytes become senescence during the AD process, exhibiting structural and functional mitochondrial impairments, reduced ATP generation, diminished antioxidant capability, and increased ROS production [39, 40, 41]. These findings suggest that aged astrocytes with defective mitochondrial function might serve as an initial trigger for neurodegeneration.

Transcellular mitochondrial transfer is a recently discovered form of cell–cell communication and cellular repair [42, 43]. Intercellular mitochondrial transfer also takes place under various pathological conditions, such as obesity, glioblastoma, and acute stroke, playing a positive role in the survival of recipient cells [13, 44, 45]. Internalization of neuronal mitochondria has been significantly increased in astrocytes of both human and mouse AD brains [15]. Our studies confirmed defects in neuronal and astrocyte mitochondria in AD mice. We further found that astrocyte mitochondria with poor quality can be transferred to neurons in 5 × FAD mice, potentially exacerbating oxidative damage to neurons and Aβ accumulation. This conclusion is also supported by in vitro experiments demonstrating that poor‐quality mitochondria isolated from the supernatant of aged astrocytes induced by Aβ_1–42_ or long‐term culture lead to neurite atrophy in cultured neurons. These findings indicate that the transfer of mitochondria between neurons and astrocytes plays distinctive roles among different disease models. Merely improving transcellular transport, without enhancing mitochondrial quality, may be insufficient to combat AD.

Exercise exerts neuroprotective effects through various mechanisms, including neurogenesis, synaptogenesis, autophagy, and enhancing neurotrophic factor production [46, 47, 48, 49, 50]. Consistent with this, low‐level physical activity has been identified as a risk factor for AD [51]. There is also substantial evidence linking oxidative stress to AD [52]. Importantly, oxidative stress not only promotes Aβ production but also impedes its clearance [53, 54]. Aerobic exercise can counteract oxidative damage in various cell types including neurons and astrocytes [55]. Mitochondria are the primary organelles responsible for exercise‐induced mitigation of oxidative stress, as exercise stimulates mitochondrial biogenesis, autophagy, and mitophagy [30, 56, 57]. In this study, we demonstrated that aerobic exercise enhances mitochondrial quality in astrocytes of AD mice and increases the efficiency of mitochondrial transfer from astrocytes to neurons, thus mitigating oxidative stress in neurons and reducing the deposition of Aβ plaques. This discovery enhances our understanding of the neuroprotective mechanisms of exercise.

It is worth noting that there exists a bidirectional connection of mitochondria between neurons and astrocytes [58]. A recent study has unveiled that neuron–astrocyte transmitophagy, the transfer of mitochondria to neighboring cells specifically for degradation [59], is increased in 5 × FAD mice at the age of 6 months onward [15]. Future research is needed to determine whether aerobic exercise mediates the transfer of mitochondria from neurons to astrocytes.

Moreover, this study suggests that the beneficial effect of aerobic exercise on astrocyte–neuronal mitochondrial transfer depends on astrocyte CD38 expression. CD38 plays a crucial role in mediating mitochondrial release and also enhances the expression of the *O*‐GlcNA cycle [60]. It has been reported that the *O*‐GlcNA cycle is involved in maintaining extracellular mitochondrial function during and after intercellular mitochondrial transfer [60]. CD38 also serves as an ADP‐ribose cyclase that produces cyclic ADP‐ribose (cADPR), leading to the release of Ca^2+^. Activation of CD38 enhances mitochondrial release in astrocytes, while the use of an intracellular Ca^2+^ chelator or CD38 knockdown reduces mitochondrial release in astrocytes [61]. Furthermore, the CD38‐cADPR‐Ca^2+^ signaling pathway mediates the release of extracellular vesicles containing mitochondria from astrocytes, rescuing neurons from apoptosis after oxygen–glucose deprivation [60]. Additionally, the CD38‐cADPR signaling pathway is involved in mitochondrial internalization, catalyzing the cyclization of extracellular nicotinamide adenine dinucleotide (NAD^+^) into intracellular cADPR, triggering the release of Ca^2+^, and promoting cytoskeleton remodeling, thus facilitating the internalization of extracellular mitochondria [62]. Collectively, these results suggest that CD38 plays a pivotal role in the entire process of intercellular mitochondrial transfer, encompassing release, extracellular transport, and internalization.

CD38 is widely expressed in various brain cell types including neurons, astrocytes, and microglia [13]. It has been reported that hippocampal astrocytes downregulates the expression of CD38 in 5‐month‐old APP/PS1 mice. Moxibustion treatment improves CD38 expression in these AD mice, contributing to the promotion of astrocyte–neuron interaction and synaptic plasticity [63]. Consistent with these findings, our study revealed that the level of CD38 was reduced in Aβ‐treated primary astrocytes and hippocampal astrocytes of 6‐month‐old 5 × FAD mice. After aerobic exercise, the low expression of CD38 in astrocytes of AD mice was restored, and the ability of astrocytes to transfer healthy mitochondria to neurons was improved, thereby reducing neuronal oxidative stress and Aβ load. Knocking down CD38 offsets the beneficial effect of aerobic exercise on AD‐like pathology. Taken together, both previous and current results emphasize CD38‐mediated mitochondrial transfer as a potential target for combating the occurrence of AD.

However, it is worth noting that a contrary view suggests that the upregulation of age‐related CD38 expression may be the primary cause of mitochondrial dysfunction [64]. CD38 functions as a NADase and consumes a substantial amount of NAD^+^ [63]. Upregulation of CD38 has been identified as a key regulator involved in NAD^+^ decline in skeletal muscle tissue during aging [65]. CD38 gene knockout or the use of a CD38 inhibitor can counteract age‐related NAD+ decline [66, 67, 68]. Moreover, previous studies suggest that although senescent human umbilical vein endothelial cells do not exhibit high expression of CD38, they can induce CD38 expression and CD38‐NADase activity in non‐senescent cells by producing components of the senescence‐associated secretory phenotype [66]. Therefore, the interaction mechanism between CD38 and mitochondrial homeostasis in age and related neurodegenerative processes is intricate and necessitates further detailed exploration.

In addition, recent studies have reported that tunnel nanotubes (TNTs) participate in the transfer of mitochondria between cells [69]. However, the proportion of mitochondria transported through TNTs remains unclear. Several neurotoxic aggregates, including tau protein, Aβ, and α‐synuclein, can promote the formation of TNTs through specific physiological mechanisms in neurodegenerative conditions, thereby mediating the spread of neurotoxicity across cells [69]. Whether the TNT pathway is involved in mitochondrial transfer from astrocytes to neurons and its role in AD‐like pathological processes remains to be determined.

In summary, the present study has revealed that aerobic exercise not only improves the mitochondrial quality of astrocytes but also facilitates astrocyte–neuron mitochondrial transfer, thereby ameliorating AD‐like pathology. The CREB‐CD38 signaling pathway appears to be a key regulatory factor for this intercellular mitochondrial transfer. Furthermore, clinical studies are needed to further identify the contribution of aerobic exercise‐mediated mitochondrial homeostasis in the prevention and treatment of AD.

## 4 MATERIALS AND METHODS

### 4.1 Animal grouping and experimental design

Five‐ and six‐month‐old male 5 × FAD mice and age‐matched WT littermate mice with a C57BL/6 background were utilized for this study. The animals were housed in a controlled environment with regulated temperature and humidity under a 12 h light: dark cycle. All animal experiments were conducted following the guidelines for the Care and Use of Laboratory Animals of Nanjing Medical University (Approval No. IACUC‐1812054).

### 4.2 Stereotactic injection

Mice were anesthetized by intraperitoneal (i.p.) injection of a mixed solution containing ketamine (100 mg/kg) and xylazine (10 mg/kg) in saline. Subsequently, they were positioned in a stereotaxic apparatus for the microinjection of 0.8 μL of AAV‐GFAP‐mito‐timer expressed a mutant of the red fluorescent protein, dsRed, in which fluorescence shifts over time from green to red as the protein matures [27]. The injection coordinates were anteroposterior −2.0 mm, mediolateral −1.8 mm, and dorsoventral −2.4 mm, and the injection duration was set at 10 min. The injection needle was retained in place for an additional 5 min and then withdrawn slowly over the course of 5 min. For CD38 silencing, bilateral hippocampal catheter implantation was performed, and a 2‐μL mixture of siRNA with the Elite Fect‐I siRNA In vivo Transfection Kit (Changzhou Baidai Co., Ltd; Cat. #11028) was injected through the catheter every 3 days [13].

### 4.3 Aerobic exercise training

The mice underwent 4 weeks of aerobic exercise on a treadmill inclined at 10 degrees [70, 71]. During the initial 2‐day adaptation period, treadmill speed started at 8 m/min for 30 min, followed by 10 m/min for an additional 30 min. Thereafter, the daily training speed initiated at 10 m/min, increased by 1 m/min every 20 min, and reached a final speed of 13 m/min. The total exercise duration was 90 min each day, with a 1‐min break for every 5 min of exercise.

### 4.4 The Y‐maze test

The Y‐maze test was conducted as per the established laboratory protocols [72]. In the initial phase, the novel arm (NA) was blocked with a plastic sheet, allowing the mice to explore the other two arms freely for 5 min. After a 2‐h interval, the plastic sheet was removed, permitting the mice to move freely in all three arms for an additional 5 min. The percentage of time spent by the mice in the NA and the number of entries into the NA were quantified using video tracking software (TopScan, CleverSys, Inc.).

### 4.5 Novel object recognition test

The mice were then subjected to the novel object recognition test [73]. Mice were given 5 min to explore the arena and objects. An hour later, the mice were placed in the same enclosure for 5 min, with a familiar object replaced by a novel object with a different shape and color. Video tracking software (TopScan, CleverSys, Inc.) was used to record the duration of time the mice spent sniffing each object within 2 cm. The discrimination index was calculated using the following formula: Novel object/(novel object + familiar object).

### 4.6 Sample preparation

After being anesthetized by intraperitoneal (i.p.) injection of a mixed solution containing ketamine (100 mg/kg) and xylazine (10 mg/kg) in saline, the mice were transcardially perfused with ice‐cold PBS. For immunoblotting analysis, the hippocampus was rapidly dissected and stored at −80°C. For immunostaining analysis, the brains were fixed in 4% PFA overnight and subsequently dehydrated by sequential incubation in 20% and 30% sucrose solutions for 48 h at 4°C. After embedding in optimal cutting temperature compound, the hippocampal sections were coronally sliced to a thickness of 20 μm using a cryostat (Leica, CM1950, Germany). For transmission electron microscopy, the hippocampus was transcardially perfused with ice‐cold PBS followed by 4% paraformaldehyde. The dissected hippocampus was placed in a 2.5% glutaraldehyde fixative and left overnight at 4°C.

### 4.7 Transmission electron microscopy

The hippocampus was dissected and placed in a 2.5% glutaraldehyde fixative overnight at 4°C. It was then rinsed in cacodylate buffer, gently scraped, pelleted, and post‐fixed in 1.0% osmium tetroxide in cacodylate buffer for 1 h on ice. After rinsing in buffer, the samples were stabilized using a small amount of 2% agarose in PBS. Ultrathin sections were post‐stained with uranyl acetate and lead citrate. The sections were examined using a FEI Tecnai G2 electron microscope with a 120‐kV acceleration voltage (FEI Company, USA). Under transmission electron microscopy, the chromatin electron density of neuronal nuclei is low with a small amount of heterochromatin plaques. The cytoplasmic electron density of neurons is also very low and rich in plate‐like rough endoplasmic reticulum. The nucleus of astrocytes is usually irregular in shape with heterochromatin accumulation near the nuclear membrane region. The prominent feature of astrocyte cytoplasm is the presence of numerous parallel‐arranged filamentous structures [74]. As for synaptic terminals, they are spherical in shape and contain various synaptic vesicles [75]. The quantitative analysis of mitochondrial integrity was performed according to the method previously described [76]. In brief, the size of each mitochondrion was obtained by drawing the electron microscopy sagittal profile of the mitochondria. The scale was set according to the bar of the picture in ImageJ software (National Institutes of Health, USA). By using the freehand selections of this software, the outline of mitochondria was carefully drawn, and then, the circumference and area of the mitochondria were measured.

### 4.8 Primary astrocyte culture

Primary astrocyte cultures were established using cerebral cortices from neonatal mice [77]. In brief, cerebral cortices were dissected from 1‐day‐old C57BL/6J mice and mechanically dissociated in Dulbecco's modified Eagle medium (DMEM). The minced tissue underwent a 0.25% trypsin digestion for 5 min and was subsequently filtered through a 200‐mesh sieve. Following centrifugation at 300 *g* for 5 min, the resulting pellet was resuspended in DMEM (pH 7.6), supplemented with 25 mM glucose, 4 mM glutamine, 1 mM sodium pyruvate, and 10% fetal bovine serum. These cells were plated in 10‐cm cell culture dishes at a density of 6 × 10^5^ cells/cm^2^. Cultures were maintained in 10‐mL DMEM with 10% fetal bovine serum in an atmosphere of 5% CO_2_/95% air at 37°C, with the medium being replaced every 3–4 days. Astrocyte cultures at day 13 were exposed to 5 μM oligomeric Aβ_1–42_ (NJPeptide; Cat. #107761‐42‐2) dissolved in DMSO. The control group received culture medium with an equivalent volume of DMSO. Some astrocytes were repeatedly subcultured for 8 weeks to establish a replicated aging model. At this point, astrocytes almost lost their proliferative ability, manifested as over 80% of cells expressing SA‐β‐Gal [78, 79].

### 4.9 Primary neuron culture

Primary neuron cultures were prepared from the cerebral cortices of neonatal mice [80]. The cerebral cortices of 1‐day‐old C57BL/6J mice were dissected and mechanically dissociated in DMEM. The minced tissue was digested with papain for 15–20 min, followed by centrifugation at 300 *g* for 5 min. The resulting pellet was resuspended in DMEM, filtered through a 200‐mesh sieve, and subsequently centrifuged at 300 *g* for 5 min. Cells were plated on poly‐d‐lysine‐coated plates and cultured in neurobasal medium supplemented with B‐27 and 0.5 mM glutamine at a density of 2 × 10^5^ cells/mL (1 mL for 12‐well format, 0.5 mL for 24‐well format). Cultures were maintained at 37°C in a humidified chamber with an atmosphere of 95% air and 5% CO_2_. Experiments were conducted using cultures established from 7 to 10 days after seeding. For labeling, neurons were transfected with HBLV‐ZsGreen NC (Hanbio, Shanghai, China) on the 5th day, and the medium was changed 24 h post‐transfection. Green fluorescence became observable under a fluorescence microscope on the 3rd day after transfection.

### 4.10 siRNA experiments

Control siRNA and CD38 siRNA were procured from Guangzhou RiboBio Co (China). Control siRNA consisted of a scrambled sequence with no known effect on specific degradation of cellular mRNA. siRNA transfections were carried out to knock down the CD38 gene using Lipofectamine 2000 (ThermoFisher, Inc., USA), following the manufacturer's protocols. A total of 33 nM CD38 siRNAs were transfected into single‐cell suspensions of astrocytes in a six‐well plate. Total RNA was subsequently extracted from astrocytes 48 h after transfection using the Trizol reagent, following the described RNA extraction protocol. The sequences for mouse CD38 siRNAs are as follows: 5′‐GUGUACUACCAACAUUCAA‐3′, 5′‐GUGUGUCUUUAGUAGGUAU‐3′, 5′‐CCAGUUUGUGAUUGUUGA‐3′.

### 4.11 Neuronal and astrocyte co‐culture

After 72 h of CD38 siRNA transfection, astrocytes were washed with PBS three times. Subsequently, astrocytic mitochondria were stained with 100 nM MitoTracker Red CMXRos (ThermoFisher; Cat. #M7412) for 30 min. After staining, astrocytes were washed with PBS three times and subjected to a 0.25% trypsin digestion for 5 min. After centrifugation at 300 *g* for 5 min, the pellet was resuspended in neurobasal medium (ThermoFisher; Cat. #21103049) and added to the neuronal culture environment. Neurons at a density of 1 × 10^5^ were seeded into each well of a 24‐well microtiter plate and co‐cultured with 1 × 10^4^ astrocytes. After co‐culturing for 24 h, the slides were incubated with 4′,6‐diamidino‐2‐phenylindole (DAPI) nuclear stain, washed three times for 5 min with PBS, and mounted with glass coverslips.

### 4.12 Isolation of mitochondria from astrocyte‐conditioned medium and treatment of neurons

To treat neurons with astrocytic mitochondria, neurons were plated at a density of 2 × 10^5^ cells/mL (0.5 mL for 24‐well format). After cultured for 6 days, we collected astrocyte‐conditioned medium and isolated mitochondria. To isolate mitochondria from Aβ‐treated astrocytes and astrocytes cultured for 2 and 8 weeks, each group of astrocytes was plated in five 10‐cm‐diameter cell culture dishes at a density of 6 × 10^5^ cells/cm^2^. Then, 50 mL of astrocyte‐conditioned medium for each dish was prepared. Cell supernatant was cleared of cellular debris by centrifugation at 1000 *g* for 10 min, followed by centrifugation at 13,000 *g* for 25 min. After each centrifugation step, approximately half of the supernatant was carefully removed before continuing centrifugation until a visible pellet was formed. The pellet containing astrocytic mitochondria was suspended in neurobasal medium and subsequently co‐cultured with primary neurons. Mitochondria from 50‐mL astrocyte‐conditioned culture treated three‐well neurons of a 24‐well plate. After co‐cultured for 24 h, neurons were prepared to measure ROS and observe astrocytic mitochondrial entering neurons.

### 4.13 ATP measurement

Relative intracellular and extracellular ATP levels were determined using the ATP‐based CellTiter‐Glo Luminescent Cell Viability kit (Promega, cat. no. G7570). In brief, for intracellular ATP levels, opaque‐walled 96‐well plates containing cell lysate (50 μL) were prepared. An equal volume of the one‐step reagent provided in the kit was added to each well and incubated for 30 min at room temperature. To measure the ATP content in extracellular mitochondria, cell supernatant was cleared of cellular debris by centrifugation at 1000 *g* for 10 min and 13,000 *g* for 25 min, and then washed once with PBS. The pellet was resuspended in 50 μL of serum‐free phenol‐free DMEM or PBS before adding an equal volume of the one‐step reagent provided in the kit, with incubation for 30 min at room temperature. ATP content was measured using a luminescence plate reader (BioTek, Cytation5, USA).

### 4.14 Measurement of mitochondrial membrane potential and ROS

Astrocytes were incubated with TMRM (1:5000; Bestbio; Cat. #BB‐41054) for 30 min or 5 μM mitochondrial superoxide indicator MitoSOX Red (ThermoFisher; Cat. #M36008) for 15 min at 37°C, washed three times with PBS, and then fixed. Slides were incubated with DAPI (Invitrogen; Cat. #D21490) nuclear stain, and micro‐images were captured using a confocal microscope (Zeiss™ LSM 510 Axiovert 200 M Confocal Laser Scanning Module) (Carl Zeiss, Jena, Germany) equipped with a Plan‐APOCHROMAT 63X/1.40 oil DIC M27 objective (Zeiss™). TMRM or MitoSOX Red was imaged using a 555‐nm LED. The fluorescence density was quantitated using ImageJ software (National Institutes of Health, USA) [81].

For flow cytometric detection of ROS, astrocytes cultured in 6‐well plates were removed from the incubator, washed with PBS, and digested with trypsin. After centrifugation at 300 *g* for 5 min, the pellet was washed with PBS at 37°C and subsequently suspended in 5 μM mitochondrial superoxide indicator MitoSOX Red (ThermoFisher; Cat. #M36008) and stained at 37°C for 15 min. Analysis was performed using FACS‐BD FACSCanto, which was detected with a 555 nm laser.

### 4.15 Measurement of astrocytic mitochondrial SOD and CAT

The SOD activity of astrocytic mitochondria was measured by the Total Superoxide Dismutase Assay Kit with WST‐8 (S0101, Beyotime), following the instructions of the manufacturer. Briefly, WST‐8 could react with superoxide radical anion (O_2_^−^) generated by xanthine oxidase to produce water‐soluble formazan dye. The reaction could be blocked by SOD because SOD catalyzes the dismutation of O_2_^−^ to produce hydrogen peroxide (H_2_O_2_) and O_2_^−^. Therefore, the production of formazan dye was negatively correlated with the SOD activity. After incubation, the absorbance was measured at 450 nm.

The CAT activity of astrocytic mitochondria was investigated by a catalase activity assay kit (S0051, Beyotime). Astrocytic mitochondria were mixed with reaction buffer followed by H_2_O_2_ addition. The decomposition of H_2_O_2_ was terminated by adding a stop solution and the remaining H_2_O_2_ was determined by using peroxidase, based on the formation of red products (*N*‐(4‐antipyryl)‐3‐chloro‐5‐sulfonate‐pbenzoquinonemonoimine), which was measured at a wavelength of 520 nm. Sample catalase enzyme activity is equal to the number of micromoles of hydrogen peroxide consumed multiplied by the number of dilutions, and the number of micromoles of hydrogen peroxide consumed is equal to the number of micromoles of residual hydrogen peroxide in the blank control minus the number of micromoles of residual hydrogen peroxide in the sample.

### 4.16 Brain tissue ROS staining

The ROS levels on the fresh frozen slices were detected using the Bestbio Tissue Section Reactive Oxygen Specimen Assay Kit (Bestbio; Cat. #BB‐470516). Briefly, after washing with the washing solution for 3–5 min, 20‐μm unfixed frozen sections were incubated in staining solution at 37°C in an incubator for 30 min. After washing with 0.01 M PBS for three times, the sections were incubated with PBS containing 1:1000 DAPI in for 5 min. After additional triple PBS wash, the sections were mounted with glass coverslips. Images were acquired using a confocal microscope (Zeiss LSM710, Germany).

### 4.17 Immunofluorescence

Frozen brain sections and coverslips cultured with cells were incubated with a blocking buffer containing 5% bovine serum albumin and 0.3% PBS‐Triton X‐100 for 1 h, then incubated with appropriate dilutions of primary antibodies overnight at 4°C. To label hippocampal neurons, sections were incubated with rabbit polyclonal anti‐microtubule‐associated protein 2 (AB5622, 1:300, Millipore). After washing with PBS, sections and coverslips were incubated with AF647‐conjugated donkey anti‐rabbit IgG (A32795TR, 1:1000, ThermoFisher) for 2 h at room temperature. To label astrocytes, sections and coverslips were incubated with rat monoclonal anti‐GFAP (ab279291, 1:500, Abcam). After washing with PBS, sections and coverslips were incubated with AF647‐conjugated donkey anti‐rabbit IgG (A32795TR, 1:1000, ThermoFisher) or AF488‐conjugated donkey anti‐rat IgG (ab150153, 1:1000, Abcam) for 2 h at room temperature. To evaluate the expression of CD38 in hippocampal astrocytes and primary astrocytes, sections and coverslips cultured with astrocytes were incubated with rat monoclonal anti‐GFAP (ab279291, 1:500, Abcam) and mouse monoclonal anti‐CD38 (ab252804, 1:100, Abcam). After washing with PBS, sections and coverslips were incubated with a mixture of AF488‐conjugated donkey anti‐rat IgG (ab150153, 1:1000, Abcam) and AF555‐conjugated donkey anti‐mouse IgG (A31570, 1:1000, ThermoFisher) for 2 h at room temperature. To evaluate the expression of SOD2 in hippocampal neurons and astrocytes, sections were incubated with a mixture containing rabbit polyclonal anti‐SOD2 (NB100‐1992, 1:500, Novus Biologicals) and rat monoclonal anti‐NeuN (ab279297, 1:500, Abcam) or mouse monoclonal anti‐GFAP (MAB360, 1:400, Millipore). After washing with PBS, sections were incubated with a mixture of AF555‐conjugated donkey anti‐rabbit IgG (A31572, 1:1000, ThermoFisher) and AF488‐conjugated donkey anti‐rat IgG (ab150153, 1:1000, Abcam) or AF488‐conjugated donkey anti‐mouse IgG (A21202, 1:1000, ThermoFisher) for 2 h at room temperature. Then, following 5‐min incubation with 1:1000 DAPI in PBS, the tissues or cultured cells were washed three times for 5 min with PBS and mounted with glass coverslips. Images were acquired using a confocal microscope (Zeiss LSM710, Germany).

### 4.18 Staining of astrocytic mitochondria

Astrocytic mitochondria were labeled using MitoTracker Red CMXRos (Invitrogen, Cat. #M7512). In brief, slides cultured with primary astrocytes were washed three times for 5 min with PBS and then stained with 100 nM MitoTracker Red CMXRos for 30 min at 37°C. Subsequently, the astrocyte culture was washed with PBS three times and fixed with 4% PFA for 15 min. Immunofluorescence for GFAP was then conducted as described above.

### 4.19 SA‐β‐gal and GFAP double staining

Slides cultured with primary astrocytes underwent a triple PBS wash followed by fixation for 15 min. Next, the slides were immersed in an SA‐β‐Gal staining solution (Cell Signaling Technology; Cat. #9860) at 37°C overnight. After a subsequent wash with PBS, the slides were blocked for 1 h with 5% blocking buffer (5% bovine serum albumin, 0.3% PBS‐Triton X‐100) at room temperature. They were then incubated with a mouse anti‐GFAP antibody (ab279291, 1:500, Abcam) at 4°C overnight. After washing with PBS, the slides were incubated for 2 h at room temperature with AF488‐conjugated anti‐rat IgG (ab150153, 1:1000, Abcam). Following 5‐min incubation with 1:1000 DAPI in PBS, the slides were washed three times for 5 min with PBS and then mounted.

### 4.20 Western blotting

Hippocampal tissues and pellets from astrocyte‐conditioned culture medium were homogenized and lysed in RIPA buffer, followed by centrifugation at 4°C and 13,000 *g* for 15 min. The extracts were separated on 10%–15% SDS‐polyacrylamide gel electrophoresis gels and subsequently transferred onto polyvinylidene fluoride membranes. After blocking in 5% defatted milk/TBST (pH 7.5, 10 mM Tris–HCl, 150 mM NaCl, and 0.1% Tween 20) for 1 h, the membranes were sectioned at appropriate molecular weights. They were then incubated at 4°C overnight with appropriate dilutions of one of the primary antibodies as follows: rabbit monoclonal anti‐CD38 (ab108403, 1:1000, Abcam), mouse monoclonal anti‐COXIV (MB8007, 1:1000, Bioworld), mouse monoclonal anti‐GAPDH (60004‐1‐Ig, 1:3000, Proteintech), rabbit polyclonal anti‐LC3 (14600‐1‐AP, 1:1000, Proteintech), rabbit polyclonal anti‐p16 (YP0827, 1:1000, Immunoway), rabbit monoclonal anti‐TOM20 (42406, 1:1000, CST), rabbit polyclonal anti‐PSD‐95 (Ab18258, 1:1000, Abcam), rabbit monoclonal anti‐p62 (Ab109012, 1:1000, Abcam), mouse monoclonal anti‐PINK1 (Ab186303, 1:1000, Abcam), rabbit polyclonal anti‐SYP (17785‐1‐AP, 1:1000, Proteintech), and mouse monoclonal anti‐β‐actin (60008, 1:1000, Proteintech). After washing with TBST, the membranes were incubated with secondary antibodies conjugated with horseradish peroxidase (1:2000; ZSGBBIO) at room temperature for 1 h and then imaged using the GE imaging system (ImageQuant LAS 4000 Mini, version 1.2).

### 4.21 Statistical analysis

The results were expressed as the mean ± SEM. When comparing two groups, a paired *t*‐test was employed. Multiple comparisons were assessed using one‐way ANOVA followed by Tukey's test, two‐way ANOVA, or repeated‐measure two‐way ANOVA followed by the Bonferroni test. Unless stated otherwise, a significance level of *p* < 0.05 was considered statistically significant. Statistical analyses were conducted using GraphPad Prism (version 8.0, La Jolla, California, USA).

## AUTHOR CONTRIBUTIONS

MX and QL conceived and designed the experiments. JC, YC, XH, HF, HS, and MY conducted biochemistry experiment and behavior tests. JC, YC, YS, and XH performed the immunostaining and cell culture. MX, QL, CS, JG, and JC wrote the manuscript. All of the authors read and approved the final manuscript.

## CONFLICT OF INTEREST STATEMENT

The authors declare that they have no competing interests.

## Supporting information


**Data S1.** Supplementary figures.

## Data Availability

The data used or analyzed during the study are available from the corresponding author upon reasonable request.
