# Supplementary material for: Aerobic exercise improves astrocyte mitochondrial quality and transfer to neurons in a mouse model of Alzheimer's disease
Source: Brain Pathol. 2024 Oct 26;35(3):e13316. doi: 10.1111/bpa.13316 (PMC11961210; doi:10.1111/bpa.13316)
Supplement: Supplementary file 1 — Data S1. Supplementary figures. [file BPA-35-e13316-s001.docx]

**
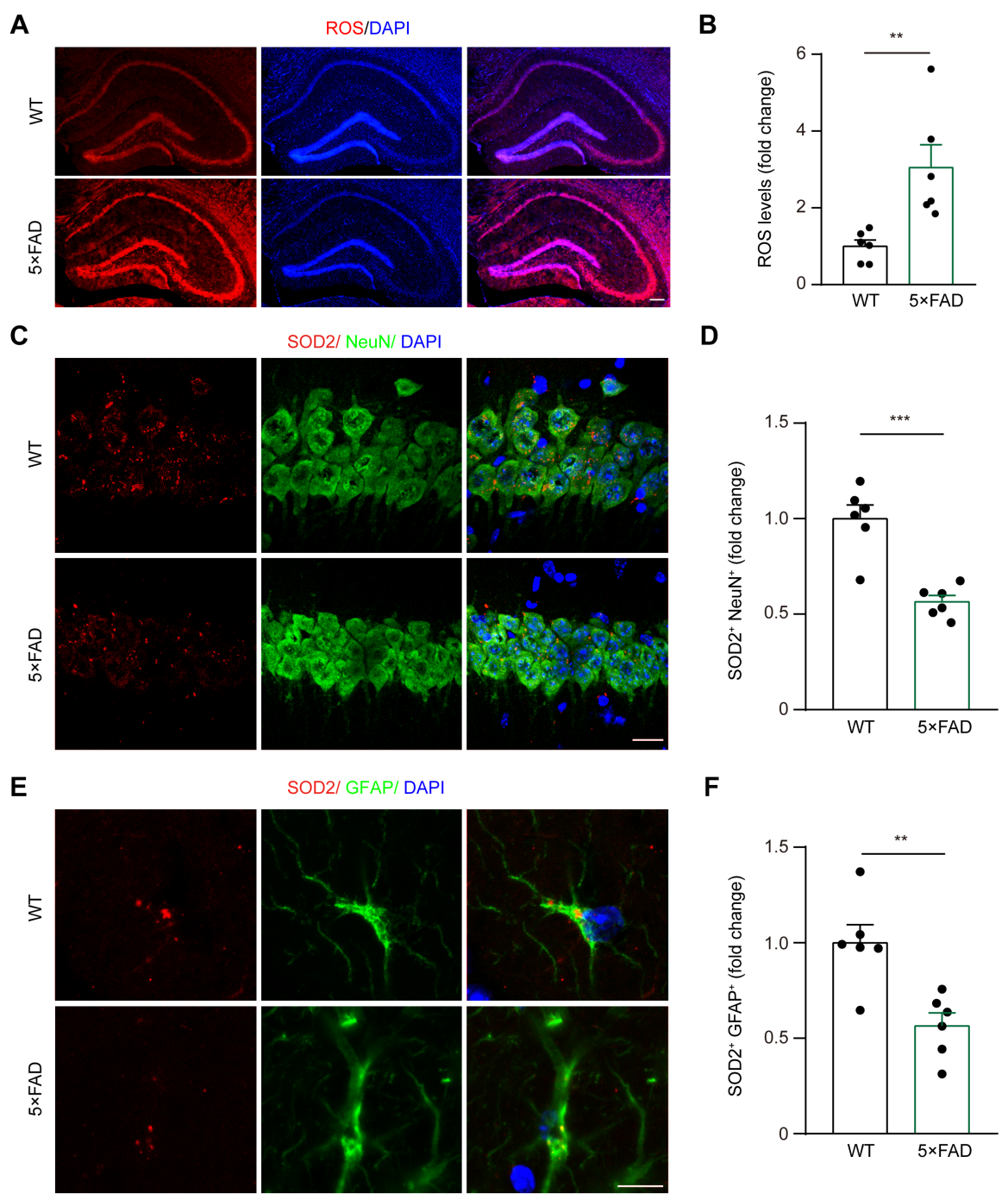
**

**Figure S1. Oxidative stress in hippocampal neurons and astrocytes of 6-month-old 5×FAD mice. (A, B)** Representative images and quantification of ROS production in the hippocampus. Scale bar: 200 μm. n = 6 mice, 5 sections per mouse. **(C, D)** Representative images of NeuN and SOD2 staining and the area of SOD2^+^ NeuN^+^ signals in hippocampal neurons of WT and 5×FAD mice. Scale bar: 20 μm. n = 6 samples. **(E, F)** Representative images of GFAP and SOD2 staining and the area of SOD2^+^ GFAP^+^ signals in hippocampal astrocytes of WT and 5×FAD mice. Scale bar: 10 μm. n = 6 samples. Paired t-test. Data represent the mean ± SEM. **P < 0.01.


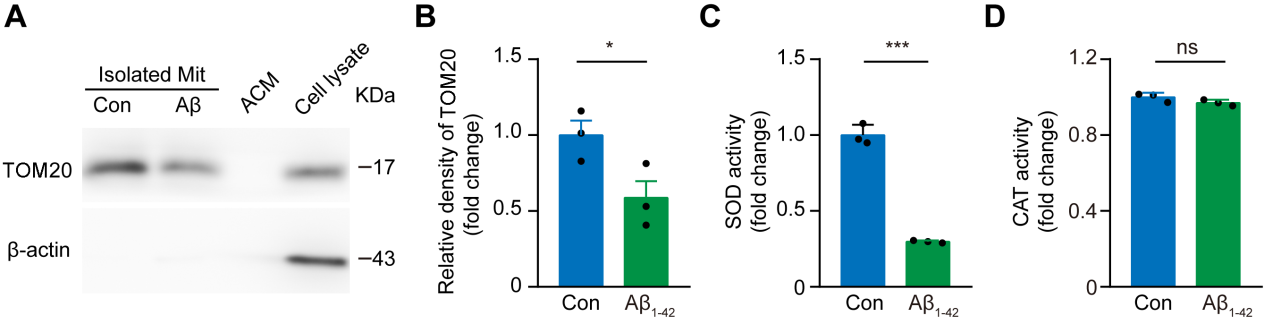


**Figure S2. Aβ-stimulated astrocytes released fewer mitochondria with low SOD1 activity. (A, B)** Western blotting and densitometry analysis of Tom20 protein levels in mitochondria extracted from control and Aβ-treated astrocyte-conditioned medium (ACM). n = 3 samples. **(C, D)** SOD1 and CAT activity in mitochondria extracted from control and Aβ-treated astrocytic ACM. n = 3 samples. Paired t-test. Data represent the mean ± SEM. *P < 0.05, ***P < 0.001; ns, no significance.

**
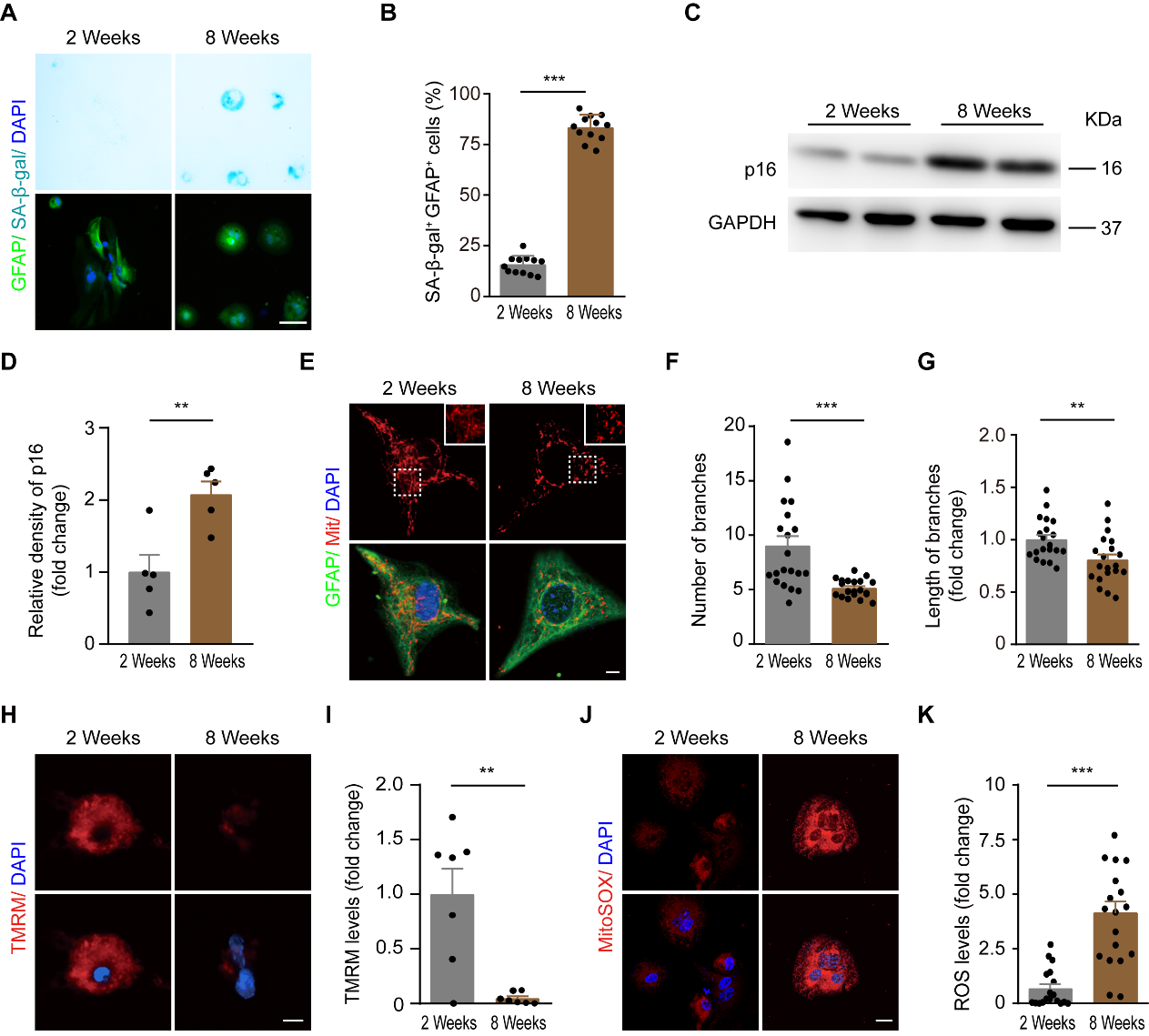
Figure S3. Mitochondrial impairment in long-term cultured primary astrocytes. (A, B)** Representative images of astrocytes cultured for 2 weeks and 8 weeks labeled with SA-β-gal and GFAP and quantification of the percentage of SA-β-gal^+^GFAP^+^ cells. Scale bar: 50 μm. n = 12 samples, 20 GFAP-positive cells per sample. **(C, D)** Western blot and densitometry analysis of protein levels of p16 in astrocytes cultured for 2 weeks and 8 weeks. n = 5 samples, triple repeats. **(E)** Representative images of GFAP and mitochondria labeled with MitoTracker Red CMXRos in astrocytes cultured for 2 weeks and 8 weeks. Scale bar: 5 μm**. (F, G)** Quantification of mitochondrial branch number and branch length. n = 20 samples, 5 GFAP-positive cells per sample**. (H, I)** Representative images and quantification of TMRM in astrocytes cultured for 2 weeks and 8 weeks. Scale bar: 10 μm. n = 7 samples, 20 TMRM-positive cells per sample. **(J, K)** Representative images and quantification of intracellular ROS production in astrocytes cultured for 2 weeks and 8 weeks. Scale bar: 20 μm. n = 18 samples, 5 images per sample. Paired t-test. Data represent the mean ± SEM. **P < 0.01, ***P < 0.001.

**
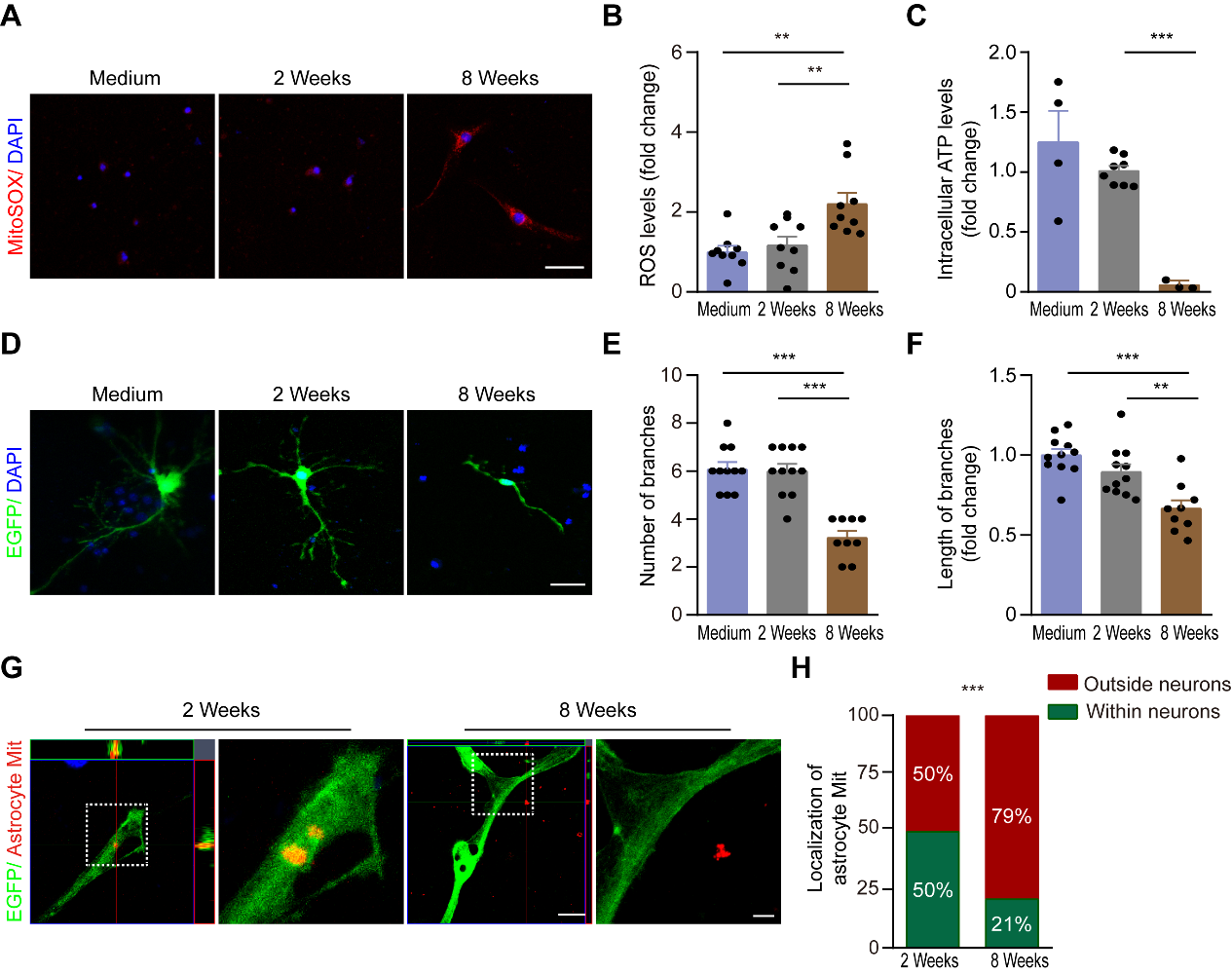
Figure S4. Mitochondria released by primary astrocytes cultured for a long time entering cultured neurons and inhibiting their growth.** **(A)** Representative images of ROS staining in neurons treated with mitochondria isolated from astrocytes cultured for 2 weeks and 8 weeks. **(B)** Quantification of ROS production in neurons treated with mitochondria isolated from astrocytes cultured for 2 weeks and 8 weeks. n = 9 samples, 10 images per sample. **(C)** Quantification of ATP levels in neurons treated with mitochondria isolated from astrocytes cultured for 2 weeks and 8 weeks. n = 4 samples for the medium, 8 for 2 weeks, and 3 for 8 weeks, triple repeats. **(D)** Representative images of cultured primary neurons treated with mitochondria isolated from astrocytes cultured for 2 weeks and 8 weeks. Scale bar: 50 μm. **(E, F)** Quantification of branch number and branch length of cultured primary neurons. n = 11 samples, 10 EGFP-positive cells per sample. **(G, H)** Representative images and percentage of mitochondria from astrocytes cultured for 2 weeks and 8 weeks (red, MitoTracker Red CMXRos) entering cultured primary neurons. n = 3 samples, 20 EGFP-positive cells per sample. Scale bar: 20 μm and 5 μm (high magnification images). One-way ANOVA with Tukey’s multiple comparisons test (Fig. S4B, C, E, and F) or Chi-square test (Fig. S4H). Data represent the mean ± SEM. **P < 0.01, ***P < 0.001.

**
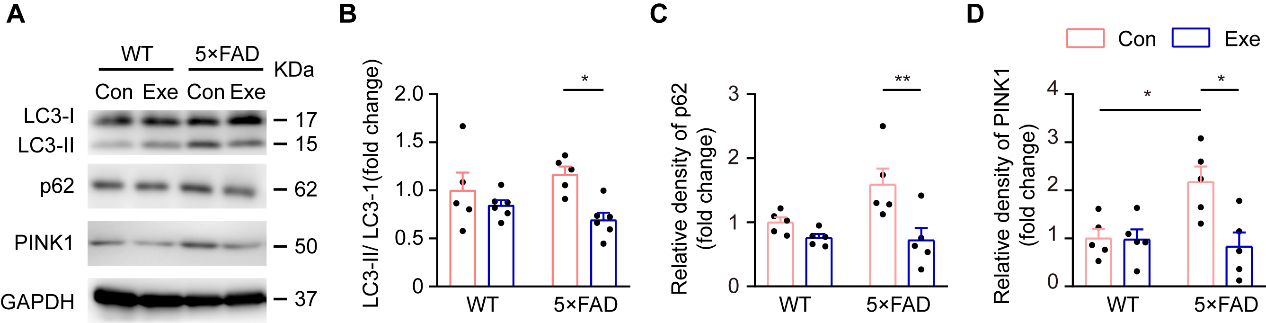
Figure S5.** **Downregulation of the makers for autophagic vacuoles in the hippocampus of 6.5-month-old 5×FAD mice by aerobic exercise. (A-D)** Western blot and densitometry analysis of protein levels of LC3, p62 and PINK1 in the hippocampus. n = 5-6 samples, triple repeats. The two-way ANOVA, Tukey’s multiple comparison test. Data represent the mean ± SEM. *P < 0.05, **P < 0.01, ***P < 0.001.


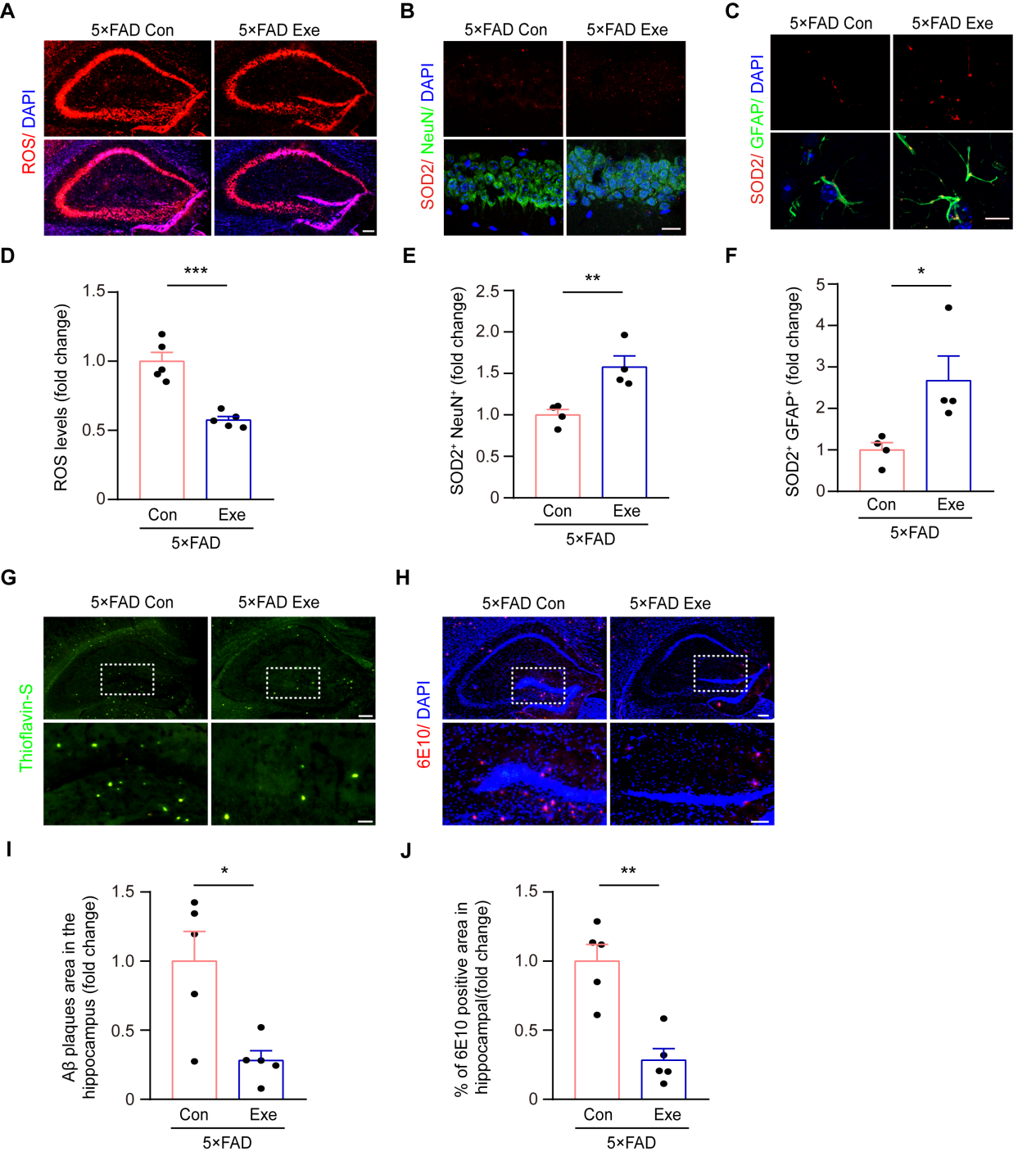


**Figure S6. Decreases in oxidative stress and Aβ load in the hippocampus of 6-month-old 5×FAD mice after aerobic exercise.** **(A, D)** Representative images and quantification of ROS production in the hippocampus. Scale bar: 200 μm. n = 5 mice, 5 sections per mouse. **(B, E)** Representative images of NeuN and SOD2 staining and the area of SOD2^+^ NeuN^+^ signals in hippocampal neurons of WT and 5×FAD mice. Scale bar: 20 μm. n = 4 samples. **(C, F)** Representative images of GFAP and SOD2 staining and the area of SOD2^+^ GFAP^+^ signals in hippocampal astrocytes of WT and 5×FAD mice. Scale bar: 10 μm. n = 4 samples. **(G, I)** Representative images and quantification of thioflavin-S positive plaques in the hippocampus. Scale bar: 200 μm and 50 μm (high magnification images). n = 5 mice, 5 sections per mouse. **(H, J)** Representative images and quantification of 6E10 positive plaques in the hippocampus. Scale bar: 200 μm and 100 μm (high magnification images). n = 5 mice, 5 sections per mouse. Paired t-test. Data represent the mean ± SEM. *P < 0.05, **P < 0.01, ***P < 0.001.


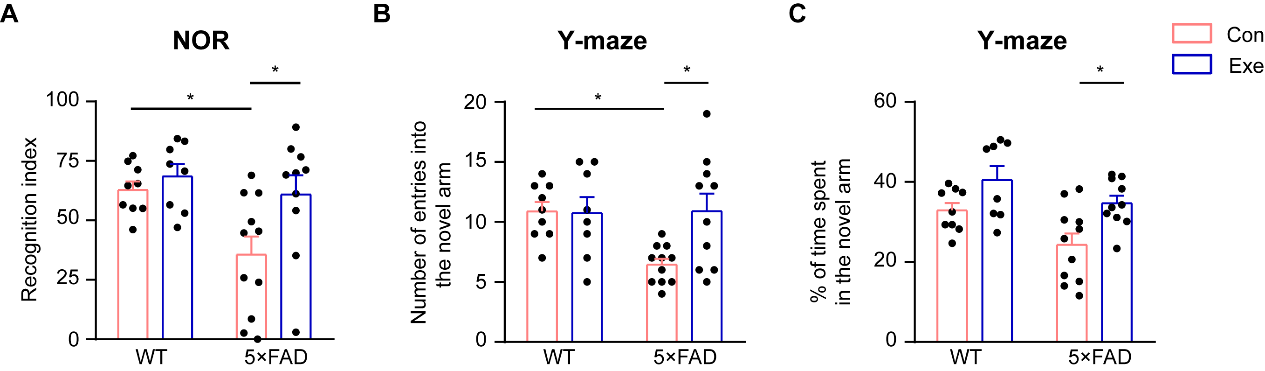


**Figure S7. Improvement of short-term cognitive function in 6-month-old 5×FAD mice by aerobic exercise. (A)** Recognition index of NORT. n = 8-11 mice. **(B, C)** The number of entries in the novel arm and percentage of time spent in the novel arm. n = 8-11 mice. The two-way ANOVA with Tukey’s multiple comparison test. Data represent the mean ± SEM. *P < 0.05.

**
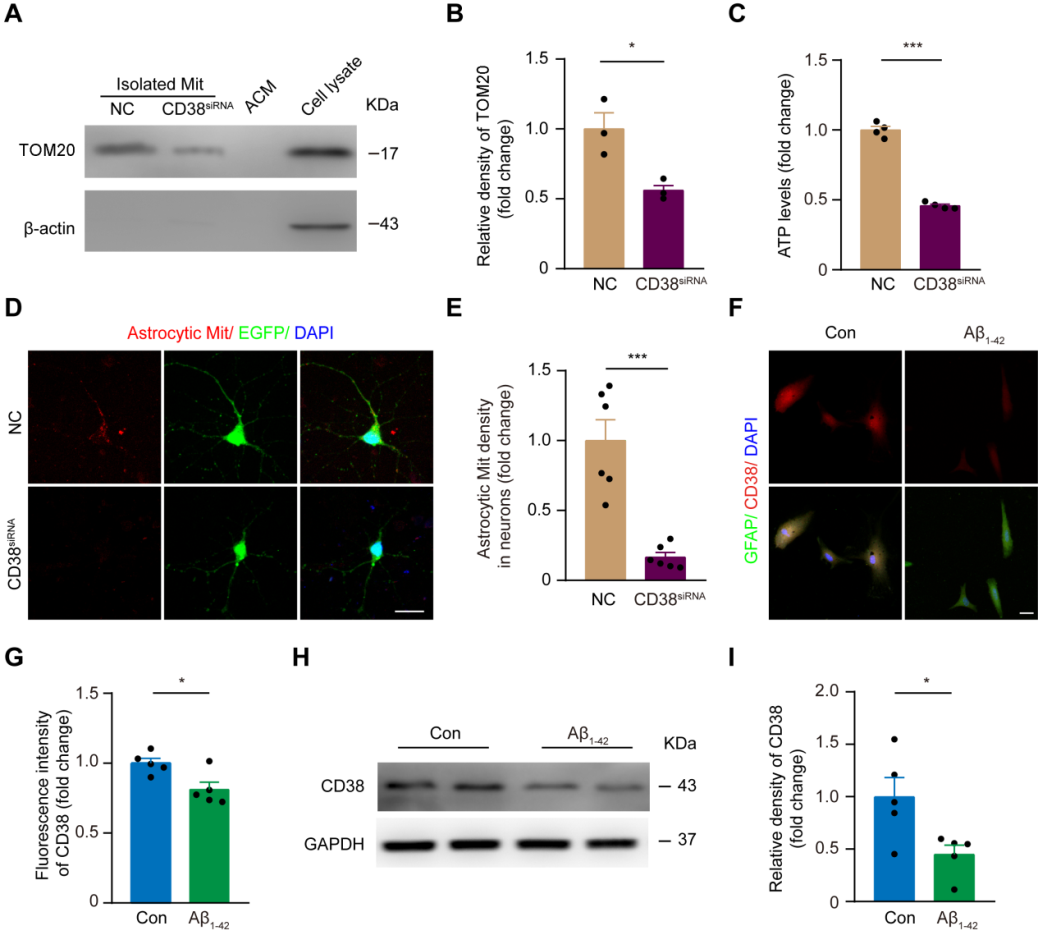
**

**Figure S8.** **Suppression of CD38 expression by siRNA inhibited astrocytic mitochondrial entry into cultured neurons. (A, B)** Western blotting and densitometry analysis of Tom20 protein levels in mitochondria extracted from astrocyte-conditioned medium (ACM) of control and CD38 ^siRNA^ astrocytes. n = 3 samples. **(C)** Extracellular ATP in isolated mitochondria from control and CD38 knockdown astrocytes. n = 4 samples. **(D, E)** Representative images of mitochondria of astrocyte which were transferred with control and CD38^siRNA^ and labeled with MitoTracker Red CMXRos in neurons and quantification of astrocytic mitochondrial density in neurons. Scale bar: 20 μm. n = 6 samples, 5 EGFP-positive neurons per sample. **(F, G)** Representative images and quantification of GFAP and CD38 staining in intact control and Aβ_1-42_-treated astrocytes. Scale bar: 20 μm. n = 6 samples, 10 GFAP-positive cells per sample. **(H, I)** Western blot and densitometry analysis of protein levels of CD38 from control and Aβ_1-42_-treated astrocytes. n = 5 samples. Paired t-test. Data represent the mean ± SEM. *P < 0.05, **P < 0.01, ***P < 0.001.


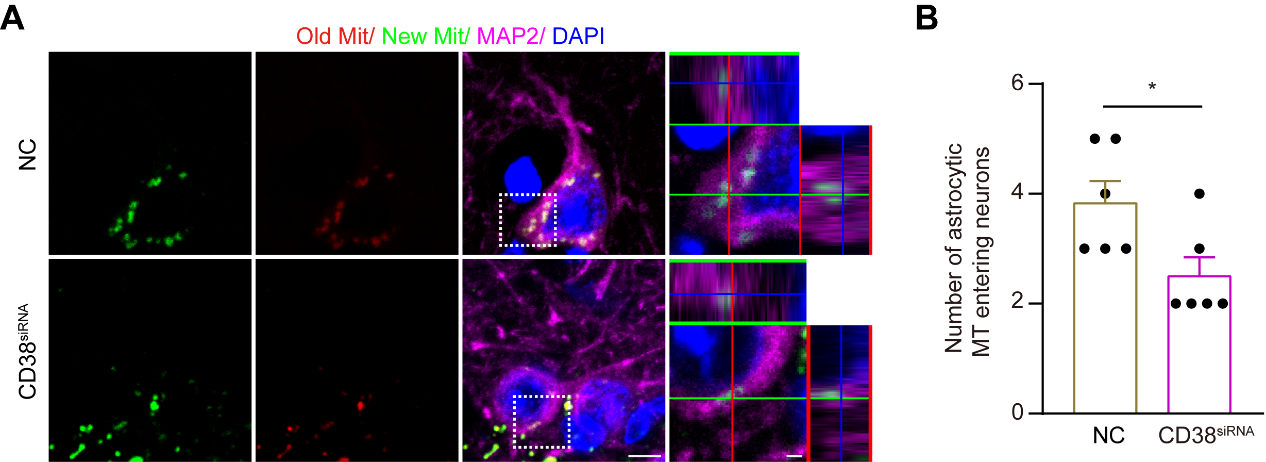


**Figure S9. Knockdown of CD38 inhibits mitochondrial transfer from astrocytes to neurons in WT mice. (A)** Representative images of astrocytic mitochondria labeled by AAV-GFAP-mito-GFP virus entering hippocampal neurons stained by MAP2 of 6-month-old WT mice treated with negative control (NC) siRNA and CD38 siRNA. Scale bar: 5 μm and 1 μm (high magnification images). **(B)** The number of hippocampal astrocytic mitochondria entering neurons of WT mice treated with NC siRNA and CD38 siRNA. n = 6 mice, 5 MAP2 neurons per mouse. Paired t-test. Data represent the mean ± SEM. *P < 0.05.
